# Integrative genomics identifies SHPRH as a tumor suppressor gene in lung adenocarcinoma that regulates DNA damage response

**DOI:** 10.1038/s41416-024-02755-y

**Published:** 2024-06-18

**Authors:** Amy L. Nagelberg, Tianna S. Sihota, Yu-Chi Chuang, Rocky Shi, Justine L. M. Chow, John English, Calum MacAulay, Stephen Lam, Wan L. Lam, William W. Lockwood

**Affiliations:** 1 Department of Integrative Oncology, BC Cancer Research Institute, Vancouver, BC Canada; 2 Department of Pathology & Laboratory Medicine, University of British Columbia, Vancouver, BC Canada; 3 Interdisciplinary Oncology Program, University of British Columbia, Vancouver, BC Canada

**Keywords:** Non-small-cell lung cancer, Cancer genomics

## Abstract

**Background:**

Identification of driver mutations and development of targeted therapies has considerably improved outcomes for lung cancer patients. However, significant limitations remain with the lack of identified drivers in a large subset of patients. Here, we aimed to assess the genomic landscape of lung adenocarcinomas (LUADs) from individuals without a history of tobacco use to reveal new genetic drivers of lung cancer.

**Methods:**

Integrative genomic analyses combining whole-exome sequencing, copy number, and mutational information for 83 LUAD tumors was performed and validated using external datasets to identify genetic variants with a predicted functional consequence and assess association with clinical outcomes. LUAD cell lines with alteration of identified candidates were used to functionally characterize tumor suppressive potential using a conditional expression system both in vitro and in vivo.

**Results:**

We identified 21 genes with evidence of positive selection, including 12 novel candidates that have yet to be characterized in LUAD. In particular, *SNF2 Histone Linker PHD RING Helicase* (*SHPRH*) was identified due to its frequency of biallelic disruption and location within the familial susceptibility locus on chromosome arm 6q. We found that low *SHPRH* mRNA expression is associated with poor survival outcomes in LUAD patients. Furthermore, we showed that re-expression of SHPRH in LUAD cell lines with inactivating alterations for *SHPRH* reduces their in vitro colony formation and tumor burden in vivo. Finally, we explored the biological pathways associated *SHPRH* inactivation and found an association with the tolerance of LUAD cells to DNA damage.

**Conclusions:**

These data suggest that *SHPRH* is a tumor suppressor gene in LUAD, whereby its expression is associated with more favorable patient outcomes, reduced tumor and mutational burden, and may serve as a predictor of response to DNA damage. Thus, further exploration into the role of SHPRH in LUAD development may make it a valuable biomarker for predicting LUAD risk and prognosis.

## Introduction

Globally, lung cancer is the leading cause of cancer mortality in both men and women, responsible for over 1.8 million deaths a year [1]. Approximately 85% of lung cancers are classified as non-small cell lung cancer (NSCLC), with lung adenocarcinoma (LUAD) the most common histological subtype, followed by squamous cell carcinoma (SCC) [2]. Incidence rates for LUAD have proportionally increased when compared to other NSCLC subtypes over the past few years. A greater understanding of the drivers of lung cancer development and progression is urgently needed in order to define new strategies for risk assessment, prevention, early diagnosis, and treatment.

The majority of lung cancers are diagnosed at an advanced stage, which limits the potential for patients to undergo possibly curative surgical resection and contributes to a 5-year survival rate of only 22% [2–4]. Advances in genomic profiling technologies have led to the identification of several genetic drivers of lung cancer and the development of drugs to target these aberrations [5]. However, while the successes of targeted therapy are numerous – including an increased quality of life for treated patients – significant challenges continue to remain. Of note, many tumors harbor mutations in uncharacterized genes or genes that are non-actionable due to difficulty of drug development. In addition, patients with lung tumors that initially respond to targeted therapies eventually develop resistance. Therefore, identification of novel genetic drivers can further elucidate strategies to better treat patients with mutations in these genes, independently or in combination with other targetable genetic drivers.

While the most well-established risk factor for lung cancer is smoking, about 28% of lung cancers diagnosed in Canada occur in people who have never smoked [2, 6]. As the mutation rate in patients who have never smoked is much lower than in smokers, there are fewer passenger gene mutations that obscure driver gene identification [7]. Many lung cancer oncogenes were originally characterized through the analysis of lung tumors from never smokers (NSs), most notably *EGFR* [8]. Thus, assessment of tumors from NS patients offers an opportunity to better understand the biology of the disease. Importantly, targets and drugs developed for the treatment of lung cancer in NSs can also be used to treat patients that have smoked with mutations in the respective drivers.

In this study, we aimed to assess the genomic landscape of LUADs in order to reveal new genetic drivers of lung cancer. We hypothesized that focusing on NS lung tumors would enrich identification of cancer drivers relative to passenger mutations, aiding in gene discovery. Using integrative genomic analyses including next generation sequencing of 83 in-house LUADs and validation in external cohorts, we identified 21 significantly mutated genes through our computational pipeline that assessed for evidence of positive selection, of which 12 were novel candidates that have yet to be characterized in LUAD. *SNF2 Histone Linker PHD RING Helicase* (*SHPRH*) was uncovered as a candidate of interest due to its frequency of double allelic disruption and location within the main lung cancer susceptibility locus on chromosome arm 6q [9]. We subsequently found that in addition to mutation, *SHPRH* copy number alterations in LUAD tumors and cell lines reduce *SHPRH* expression and that low *SHPRH* expression is associated with worse clinical outcomes in LUAD patients. Furthermore, we demonstrate that re-expression of SHPRH in LUAD cells with inactivating alterations reduces their tumorigenic potential in vitro and in vivo, highlighting *SHPRH* as a novel tumor suppressor gene in LUAD. Finally, investigation into the role of *SHPRH* expression in LUAD tumors suggests that it may confer a protective effect against DNA damaging agents. Together, this work suggests that SHPRH is a tumor suppressor gene in LUAD whose expression is associated with reduced LUAD development and more favorable prognosis and – coupled with its chromosomal location in a susceptibility locus and role in DNA damage tolerance – warrants further assessment as a potential biomarker for individuals at-risk of developing LUAD.

## Materials and methods

### Sequencing and variant calling

A panel of 15 NS LUAD tumors and matched normal lung tissues were obtained from BCCA under informed consent as previously described [10]. Whole-exome sequencing (WES) using the Illumina platform was performed at the NIH Intramural Sequencing Center (NISC) according to standard protocols and resulting BAM files were aligned to the hg19 reference genome as previously described [11, 12]. BCFTools [13], Varscan [14], and Freebayes [15] were used to make variant calls for tumor and matched-normal controls. ‘Base Quality’ was set to ≥10 and ‘Depth’ to ≥8, while all other parameters were set at default. Resulting VCF files were sorted and normalized using BCFtools [16]. Variants that were called in at least two of the three algorithms were maintained in tumors, and variants called by any one of the three algorithms were maintained in matched-normal controls. VCF files were prepared and subsequently annotated with ANNOVAR [17].

### Somatic filtering

Variants that were called by any algorithm in any matched-normal control were removed from downstream consideration of somatic alteration in tumors. Variant lists were prepared for evaluation with MutScan [18] in R using manipulation with maftools [19] and dplyr [20]. Fastq files were generated from BAM files using Samtools [21] for use in Mutscan. Variants with an allelic frequency in normal controls of 5% or more were removed from further consideration. Variants with no reads supporting the alternative allele but with more than 20 reads supporting the reference allele were removed. In addition, variants with less than twice the percentage of reads supporting the alternative allele compared to the percentage in the matched-normal controls were removed. Variants filtered by any of the above metrics within a sample were also removed from consideration across the entire sample set. Finally, variants that were annotated by ANNOVAR (avSNP150) as being in dbSNP [22] with a healthy population frequency of more than 1% were removed. Population frequency was determined by “PopFreqMax” by ANNOVAR [17], containing the maximum allele frequency from several databases including 1000 Genomes Project [23], ESP6500 [23, 24], and ExAC [25].

### Calculation of validation rate

A custom Illumina exon capture assay was designed to assess a panel of 268 genes in the 15 tumor/normal pairs analyzed by WES (above) in addition to an independent set of in-house 68 LUAD tumor and matched normal pairs. Genes selected for capture were determined through assessment of identified variants from WES and common drivers previously reported to be mutated in LUAD and other cancers. Somatic variants in genes and samples that were sequenced by custom capture were used for validation (*n* = 488). Variants were considered validated if 5% or more of the reads identified in MutScan [18] supported the alternative allele.

### Filtering of variants for biologically relevant effects

Candidate somatic mutations were filtered for functional relevance through the following sequence. First, variants that were not annotated by ANNOVAR [17] or RefSeq [26] databases as located in protein coding or splice site regions were removed. Second, variants that were predicted to be silent at the amino acid level and not predicted by SPIDEX [27] to affect splicing (annotated by ANNOVAR, |DPSI z-score| ≥ 2) were removed. Third, variants that were in genes determined not to be expressed in NS LUAD as determined by Illumina expression microarray analysis of a panel 30 BCCA samples were removed using a pipeline previously described [28]. Lastly, amino acid functional change was predicted with SIFT [29], LRT [30], MutationTaster [31], MutationAssessor [32], FATHMM [33–35], and MetaSVM [36] by ANNOVAR [17] and if at least half of the algorithms predicted that a variant was tolerable to protein function, the variant was removed, unless it was an indel or had a SPIDEX [27]| DPSI z-score|≥2.

### Copy number analysis

Copy number processing was performed as described in previous studies [10, 37]. In brief, genomic DNA from the 83 LUAD tumors and matched non-malignant lung tissues were hybridized to Affymetrix SNP 6.0 arrays according to the manufacturer’s instructions and the resulting normalization and copy number segmentation was performed using Partek Genomics Suite Software (Partek Incorporated, Missouri) with the same settings and downstream processing described previously [37]. Resulting segmentation files were used in GISTIC 2.0 [38, 39] on Gene Pattern (https://cloud.genepattern.org/gp/pages/login.jsf) with amplification threshold = 0.8, deletion threshold = −0.6, join segment size = 50, q-value threshold of 0.05, and hg19 genome build, as described previously [40]. All other parameters were set to default.

### Analysis of external LUAD datasets

Copy number status, methylation status (HM450) and gene expression data (RNA Seq V2 RSEM) for 230 LUAD tumors [41] were downloaded from the MSKCC cBioPortal (www.cbioportal.org) [42, 43] Affymetrix U133 Plus 2 expression data for 58 LUAD tumors and exfoliated bronchial cells of 67 lung cancer–free individuals obtained during fluorescence bronchoscopy were obtained and processed as previously described [44, 45]. Copy number status and mRNA expression for 74 LUAD cell lines were downloaded from the Cancer Dependency Map portal (https://depmap.org/portal/) [46, 47]. The Kaplan-Meier (KM) plotter (https://kmplot.com/analysis/index.php?p=service&cancer=lung) [48] was used to evaluate the correlation between SHPRH mRNA expression (Affymetrix microarray) and OS and between *SHPRH* mRNA expression and PFS in LUAD patients with data downloaded from Gene Expression Omnibus (GEO) database on 14-Mar-2024. For all LUAD patients, a sample size of *n* = 672 for OS and *n* = 528 for PFS was used, with *n* = 140 for the NS subset. For all SCC patients, a sample size of *n* = 527 for OS and *n* = 220 for PFS was used. Samples were divided into high expression and low expression groups based on a median cutoff or lower quartile cutoff of *SHPRH* expression. Hazard Ratio (HR), 95% confidence interval (CI), and log-rank P-value were then determined and displayed on KM survival plots. A continuous phenotype Gene Set Enrichment Analysis (GSEA) was performed on mRNA expression data obtained for 510 LUAD tumors from the TCGA PanCancer Atlas [49] dataset obtained from cBioPortal [42, 43] using the GSEA software v4.3.2 [50, 51] and the MSigDB Hallmark gene sets [52]. The same PanCancer Atlas LUAD dataset was also used to assess the impact of SHPRH inactivation (copy number loss or mutation) on Tumor Mutational Burden, Mutation Count, and Fraction of Genome Altered for samples with available clinical information downloaded from cBioPortal. All TCGA PanCancer Atlas studies within cBioPortal were also assessed for SHPRH mutations and plotted to determine recurrent mutations that occur across cancer types. Lastly, LUAD datasets within cBioPortal with sequence and/or copy number information for SHPRH were assessed to determine alteration frequency of SHPRH in comparison to other previously established tumor suppressor genes.

### Cell lines and culture conditions

All cell lines were obtained from American Type Tissue Culture (ATCC), Manassas, VA and were grown at 37 °C with 5% CO_2_. HPL1D, NCI-H1395, NCI-H2347, NCI-H2009, PC9 and A549 were grown in RPMI-1640 medium (Gibco, 11875119) supplemented with 10% FBS (Gibco, 123483020) and 1% Pen/Strep (Gibco, 15140-122). NCI-H1623 was grown in RPMI-1640 medium supplemented with 5% FBS and 1% Pen/Strep. 293T cells were grown in DMEM medium (Gibco, 12430062) supplemented with 10% FBS. For experiments including doxycycline (dox)-inducible constructs, cells were grown in RPMI-1640 medium with 10% tetracycline (tet) system approved tet-free FBS (Clontech, 631101) and 1% Pen/Strep. Cells were regularly checked via polymerase chain reaction [53] for mycoplasma contamination and were confirmed to be mycoplasma negative. Where indicated, doxycycline hyclate (Sigma-Aldrich, D9891) was added to cell culture medium at 100 ng/mL.

### Plasmids and generation of stable cell lines

pBABE GFP was gifted from William Hahn (Addgene #10668). A sequence-verified human SHPRH cDNA clone from the Mammalian Gene Collection (MGC) was obtained from Horizon Discovery (Dharmacon, MHS6278-211690436). SHPRH was amplified using the following primer sequences: 5’-CACCGGGTCTTTCGGAAGATAACTGA-3’ and 5‘-AAGCCACTGTATAACCAGAACAA-3’. GFP and SHPRH were purified and subcloned into pENTR/D-TOPO using the pENTR™/D-TOPO™ Cloning Kit (Invitrogen, K240020). SHPRH was then cloned by Gateway LR Clonase II enzyme reaction (Life Technologies, 11791020) into pInducer20 (gift from Stephen Elledge, Addgene #44012) [54]. Correct sequence insertions were verified by Sanger sequencing.

For knockout of SHPRH, sgRNA sequences targeting SHPRH (sgSHPRH.ex4 5’-ATGCTGGACATCCACTTGGA-3’) were cloned into the lentiCRISPRv2 plasmid (gift from Feng Zhang, Addgene #52961) using the Zhang Lab Target Guide Sequence Cloning Protocol obtained from Addgene [55, 56]. Undigested lentiCRISPRv2 plasmid lacking a sgRNA insert was used as a control. Sanger sequencing was used to verify correct sequence insertions.

293T cells, psPAX2 (Addgene #12260) and pMD2.G (Addgene #12259) were used to generate and package lentivirus with either the pInducer20-GFP (TetO GFP) or pInducer20-SHPRH (TetO SHPRH) recombinant lentiCRISPRv2 vectors (SHPRH KO) or the undigested control (EV) vectors. NCI-H1395, NCI-H2009, NCI-H1623, PC9, HPL1D and A549 cells were transduced with lentivirus and Lipofectamine 2000 (Life Technologies, 11668019).

Antibiotic selection with G418 was performed (Gibco, 10131027) at 375 µg/mL (NCI-H1395 and NCI-H2009), 500 µg/mL (NCI-H1623 and PC9), or 7 mg/mL (HPL1D) for 2 weeks to generate stable polyclonal cell lines. A549 was selected with 0.75 µg/mL puromycin (Sigma, 540222) for 3 days. For monoclonal populations, polyclonal cells were sorted into 96-wells, then expanded into single-cell derived colonies. Sanger sequencing was used to verify correct sequence insertions.

### Lysate generation and Western blot analysis

Cell and tumor lysates were generated using RIPA lysis buffer (Thermo Scientific, 89901) with Halt^TM^ protease and phosphatase inhibitor cocktail (Thermo Scientific, PI78444). Where indicated, cells were treated with 100 ng/mL doxycycline for 3 days prior to lysate collection. Lysates were sonicated and BCA protein assay kit (Thermo Scientific, PI23225) was used to determine protein concentration. Samples were denatured by boiling in loading buffer (Thermo Scientific, NP0007) and reducing agent (Thermo Scientific, NP0009). 20 ug of lysates were loaded on 4-12% Bis-Tris NuPage Protein Gels (Thermo Scientific, NP0335), run in MOPS SDS buffer (Thermo Scientific, NP000102), transferred to PVDF Immobilon (Millipore, IPVH00010) in transfer buffer at 70 V for 2 h, and blocked in TBS-T (TBS, 0.1% Tween20) and 5% skim milk. Membranes were immunoblotted overnight at 4 °C in TBS-T with 5% BSA (Sigma, A9647) with the following primary antibodies: SHPRH (1:1000, Sigma SAB2105240, only used for A549 KO validation) (1:1000, abcam, ab80129), GFP (1:1000, Cell Signalling Technology, 2956S), and β-Actin (1:3000, Cell Signalling Technology, 12620S). They were then incubated in HRP-linked secondary anti-rabbit IgG (1:10000, Cell Signalling Technology, 7074S) in TBS-T with 5% skim milk for 1 h at room temperature. Protein expression was visualized using either SuperSignal^TM^ West Pico Plus (Fisher Scientific, PI34580) or Femto (Fisher Scientific, PI34096) Chemiluminescent Substrate on the ChemiDocMP Gel Imaging System (BioRad). Quantification of protein band density was done using ImageJ, and calculated relative to control conditions.

### Cell viability assays

5 × 10^3^ cells were seeded in a 96-well plate in tet-free media or dox-containing (100 ng/mL) media and left to grow for 92 h, after which alamarBlue cell viability agent (Life Technologies, Dal1100) was added at a 10% concentration. Cells were incubated with alamarBlue for 4 h. Fluorescence (Excitation: 540 nm, Emission: 590 nm) was measured using a Cytation 3 Multi Modal Reader with Gen5 software (BioTek).

### Clonogenic assays

Cells were treated with or without 100 ng/mL dox 3 days prior to seeding. 200 (PC9), 700 (HPL1D), 1 ×10^3^ (NCI-H2009), or 1 ×10^4^ (NCI-H1395 and NCI-H1623) cells were seeded in 6-well plates in tet-free media or dox-containing media and left to form colonies. For the clonogenic survival assays, cells were also treated with various concentrations of MMS (v/v) (Sigma, 129925), etoposide, (Selleckchem, S1225), cisplatin (Selleckchem, S1166), or olaparib (Selleckchem, S1060), alongside their vehicle controls (media only for MMS; DMSO (Fisher Scientific, BP231) for etoposide and olaparib; and saline (0.9% NaCl solution) for cisplatin). Cells were plated in triplicate. Media was changed every 3–4 days. 10 days (PC9, HPL1D, and NCI-H2009), 2 weeks (NCI-H1395) or 4 weeks (NCI-H1623) post-seeding, colonies were stained using a 20% Methanol and 0.1% Crystal Violet (Sigma, HT90132) solution and left to dry. Colonies were then destained using 10% Glacial Acetic Acid and the absorbance was read at 590 nm using the Cytation 3 Multi Modal Reader with Gen5 software. For each independent experiment, absorbance values were averaged between the triplicates of each condition and calculated relative to the GFP no dox control.

### Soft agar colony formation assays

Cells were treated with or without 100 ng/mL dox 3 days prior to seeding. In 12-well plates, a bottom agar layer containing RPMI-1640, 10% tet-free FBS, 1% Pen/Strep, and 0.6% Low Melting Point (LMP) agar was used. 1 × 10^3^ (PC9 and NCI-H2009), 5 ×10^3^ (NCI-H1395), 7 ×10^3^ (HPL1D), or 1 × 10^4^ (NCI-H1623) cells were suspended in a top layer containing 0.3% LMP agar instead. Where indicated, 100 ng/mL of dox was added to both the top and bottom layers. Cells were plated in triplicate and hydrated with 30 µl of tet-free or dox-containing media every 3-4 days. After 2 weeks (PC9), 3 weeks (HPL1D), 4 weeks (NCI-H1395), or 5 weeks (NCI-H2009) formed colonies were stained with 5 mg/mL of MTT (3-(4, 5 – Dimethyliazol-2-yl)-2,5-Diphenyltetrazolium Bromide) for 30 minutes and the number of colonies was determined from scanned images of the plates using ImageJ software find maxima tool. For each independent experiment, colony counts were averaged between the triplicates of each condition.

### In vivo mouse xenografts

Tumor formation was assessed in male JAX-NRG (NOD-*Rag1*^*null*^ *IL2rg*^*null*^) mice kept on a normal or dox (Envigo Teklad, TD.130141) diet. NCI-H1395 and NCI-H2009 cells were treated with or without 100 ng/mL dox 3 days prior to injection. A549 cells were kept in normal cell culture medium. 1 × 10^6^ cells in 100 µl of PBS (for NCI-H1395) or serum-free RPMI-1640 medium (for NCI-H2009) were alternately injected subcutaneously into the left and right flanks of 8-to-10-week-old mice obtained from the BCCA Animal Resource Centre. Cages were randomly assigned to either normal or dox diet. Group allocations were not blinded and exclusion criteria was pre-established prior to data collection which included injection error or humane endpoint due to ulceration. Mice that did not reach experimental endpoint were excluded from data analysis. Mean ± SEM and results from Student’s paired *t* test are shown. All paired measurements were assumed to be random, representative samples with normal distribution and homogenous variance, allowing the use of Student’s paired *t* test. Tumor volume was measured twice a week following injection. Experiments were terminated once tumor volume reached 2 cm^3^ for a given mouse in the cohort. Tumors were harvested, weighed, and homogenized for lysate collection.

### Neutral comet assay

NCI-H1395^SHPRH-DEL^ cells were treated with 100 ng/mL dox for 3 days. Following this, cells were treated with 0.01% MMS (v/v) or mock-treated (media change only) for 4 h. Cells were then collected and the neutral comet assay was performed according to the manufacturer’s instructions for the CometAssay Reagent Kit for Single Cell Gel Electrophoresis Assay (R&D Systems, 4250-050-K). Comets were stained with 16 µg/mL Propidium Iodide (Thermo Scientific, P1304MP) and imaged on a LeicaDMi8 microscope at 10X magnification. Tail moment was obtained using an ImageJ plugin that has been previously described [57, 58]. Measurements were blind reviewed by individuals without prior knowledge of sample order. 70 events per condition were analyzed from each independent experiment.

### RNA sequencing analysis

1 ×10^6^ NCI-H1395 TetO cells were seeded in triplicate into 10 cm plates. The following day, media was changed and the cells were treated for 72 h with or without 100 ng/mL of dox. Total RNA was extracted using the RNeasy Mini kit (Qiagen) according to the manufacturer’s protocol. Sample quality assessment, library preparation, and RNA sequencing (RNASeq) was performed by GENEWIZ™ from Azenta Life Sciences (New Jersey, USA). RNA Integrity Numbers (RIN) for all samples were ${{{{{\boldsymbol{\ge }}}}}}$9.5. Quality Control was performed using FastQC [59] and Illumina adapters were trimmed using CutAdapt [60]. Trimmed read files were aligned to the GRCh38.p14 (Release 44) reference genome using Salmon [61]. R Statistical Software v. 4.3.2 [62] was used for downstream analysis. A matrix of read counts was generated via the tximport v.1.30.0R package [63]. Normalized TPM values were then log(n + 1) transformed using the dplyr v.1.1.4R package [20], and a differential expression score for each gene was calculated for the +dox vs -dox state for both the TetO-GFP and TetO-SHPRH cell lines individually as previously described [64, 65]. GSEA was then performed on the resulting gene list for each cell line using the preranked GSEA function and the H: hallmark gene sets collection from the Molecular Signature Database (MSigDB). Genes were ranked according to their differential expression scores and GSEA was run with default settings. Gene sets significantly enriched in the +dox state were then cross-referenced between the TetO-SHPRH and TetO-GFP cell lines and only those found specifically in TetO-SHPRH considered.

### Statistical analysis

Cox univariate regression model was used for survival analysis by KM plotter, with a log rank *p*-value ≤ 0.05 being considered to be statistically significant [48]. Data obtained from the TCGA Lung Adenocarcinoma Dataset [41], Cancer Dependency Map portal (https://depmap.org/portal/) [46, 47], and biological data was plotted and analyzed using GraphPad Prism version 8.2.1 (GraphPad Software, San Diego, CA, USA). See figure legends for the type of statistical test used for each experiment and the number of independent replicates that were performed. For cell-based experiments, data from 3-4 independent replicates were collected, and Mean ± SEM of independent replicates and results from Student’s *t* test are shown. For in vivo experiments, based on observations from previous studies, a minimum sample size of 5 was estimated to be necessary and Mean ± SEM of independent replicates and results from Student’s paired *t* test are shown. For both, replicates were assumed to be random, representative samples with normal distribution and homogenous variance, allowing the use of Student’s *t* test. *P* values ≤ 0.05 were considered statistically significant, with *p ≤ 0.05, **p ≤ 0.01, and ***p ≤ 0.0001. For statistical analyses performed by the GSEA software v4.3.2, a NOM *p*-value < 0.05 and FDR *q*-value < 0.25 was considered to be significant.

## Results

### Sequencing of LUADs from NSs reveals somatic variants with evidence of selective pressure

LUADs and matched normal lung tissues from 15 individuals without a history of tobacco use were collected under informed consent as previously described [10] (Supplementary Table 1). WES was performed with Illumina technology (Supplementary Table 2) and variant calling with three different algorithms was performed to identify somatic candidates (Supplementary Fig. 1A). These were then filtered against matched-normal tissues from the patients, MutScan reads, and healthy control population databases (e.g. dbSNP) to further confirm that the mutations occurred somatically. After filtering, 10,179 somatic mutations remained across all 15 tumors. The mutations in the NSs consisted of less C->A transversions than smokers, which is consistent with the known carcinogenic effects of tobacco smoke (Supplementary Fig. 1B, C). The 15 tumors had an average of 120.3 coding variants per tumor (Range 15-519, Supplementary Fig. 1D). Overall, the LUADs from our dataset had a functional mutational burden (median = 28 per tumor) similar to LUADs from NS in The Cancer Genome Atlas (TCGA, median = 21 per tumor), much lower than the burden observed in LUAD from individuals with a history of tobacco smoking (median = 64 per tumor, Supplementary Fig. 1E).

**Fig. 1:**
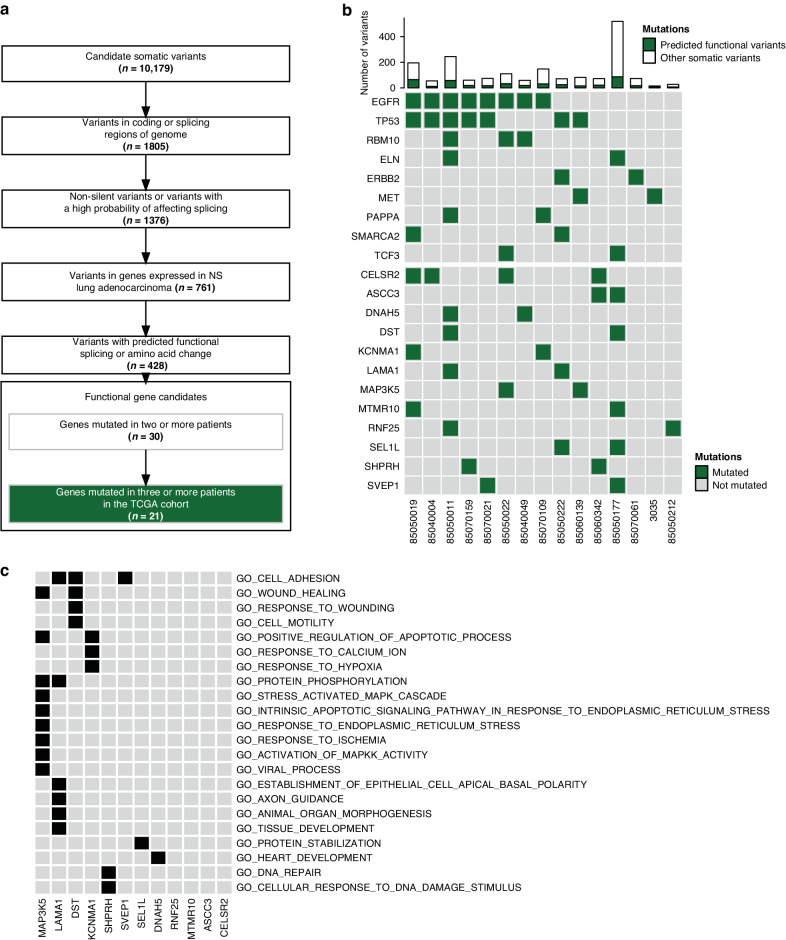
Functional analysis and filtering identifies candidate genes altered in NS LUAD. **a** Workflow used to predict variants in genes undergoing positive selection in LUAD. **b** A summary table illustrating the presence of genetic mutations in the 21 genes passing functional and prevalence filtering across the 15 tumors. The top bar illustrates total somatic mutations across the cohort with retained functional mutations shown in green. **c** GO enrichments present in 3 or more samples that have members in novel candidate genes. Black boxes illustrate the gene being annotated with GO.

We next filtered the identified somatic mutations to remove variants in genes that may be mutated without functional consequence, as these would be predicted to be probable passenger mutations. Following removal of non-coding and silent variants, we searched for variants in genes that are expressed in LUADs from NSs, as it has previously been demonstrated that genes not expressed in specific cancers have a higher mutation rate due to limited selective pressure [28] (Fig. 1a). Finally, amino acid functional prediction by various algorithms [29–36] as annotated through ANNOVAR [17] was assessed and only variants with at least half of the algorithms predicting a deleterious consequence to protein function were considered as a functional amino acid substitution. Variants that were either indels, functional amino acid substitutions, or predicted to affect splicing (SPIDEX|DPSI z-score|≥2) were considered candidates, resulting in 428 variants with a predicted functional consequence across the 15 samples (Fig. 1a). The median per-sample validation rate was 87% (Supplementary Table 3), as determined by a secondary custom capture panel of select genes (see Methods).

To implicate new candidates involved in tumorigenesis, we next assessed the frequency of gene mutation, identifying 30 genes mutated in two or more of the tumors (Fig. 1a). Mutation frequency across the TCGA LUAD cohort [41] (*n* = 230) was also assessed to further infer prevalence in LUAD tumors from smokers and NSs. Only genes that were mutated in three or more TCGA LUADs were considered, resulting in a total of 21 candidate genes (Fig. 1a). These genes consisted of known and suspected drivers of lung cancer, as well as genes that have yet to be functionally validated or characterized in this disease. For the purposes of identifying genes that are novel and therefore attractive targets to further characterize for a role in lung cancer biology, known drivers were defined as those that are present in the COSMIC as a census gene [66] and present in lung cancer (*EGFR, TP53, ERBB2, MET, RBM10, TCF3, ELN*), or those genes that have a confirmed functional role or evidence of therapeutic potential as evaluated in lung cancer (*PAPPA, SMARCA2*). All 21 genes are shown in Fig. 1b, with the 12 novel candidates segregated in the bottom of the panel. The 12 novel candidates are: *CELSR2, RNF25, KCNMA1, SHPRH, DNAH5, MAP3K5, ASCC3, SVEP1, DST, MTMR10, SEL1L* and LAMA1. Hypergeometric Gene Ontology (GO) enrichment analysis of these candidates was performed for each NS LUAD tumor sample to determine if GOs were recurrently enriched across samples, revealing a range of cellular functions with which some of the candidates were annotated, while others have less defined roles to date (Fig. 1c).

### Penetrance of candidate gene disruption and assessment of two-hit frequency in expanded lung cancer datasets

**Fig. 2:**
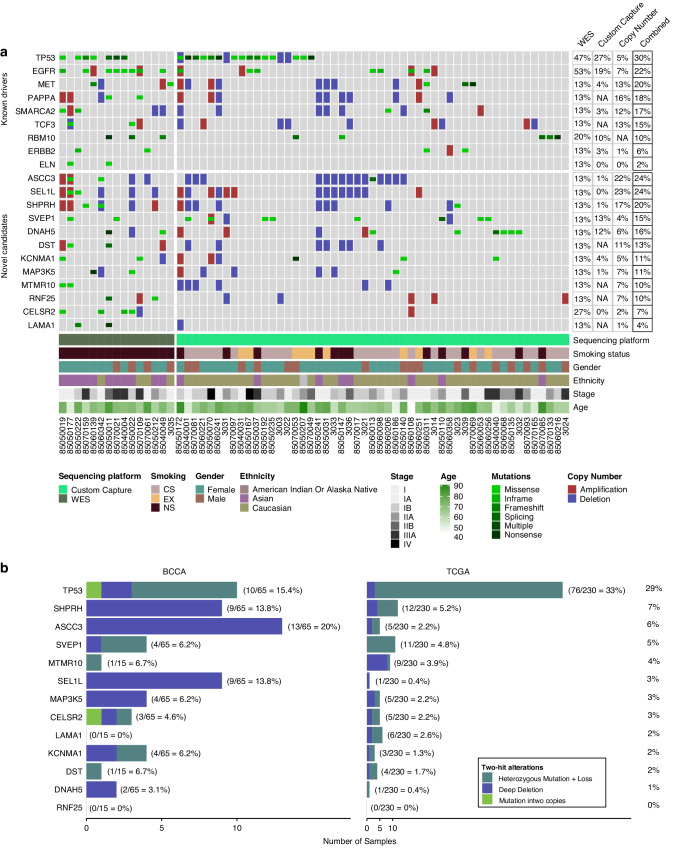
Genomic alterations and prevalence of candidate driver genes across expanded panel of LUAD tumors and assessment of two-hit frequency. **a** Oncoprint represents integration of genetic mutation and copy number status of the 12 identified candidates (bottom) and 10 known drivers (top) across 83 LUAD tumors. Samples with no alterations in the 21 genes are not shown. Frequency of disruption across the dataset is shown in the right panel and patient demographics are illustrated on the bottom. **b** Frequency of two-allele disruption in candidates in BCCA and TCGA cohorts, including homozygous mutations, deep deletions, and patients with a heterozygous mutation and loss. TP53 is shown as a control.

Genes important for tumorigenesis are typically altered by multiple mechanisms and we have previously shown that integrating different dimensions of genomic data can aid in cancer gene discovery [10, 45, 67]. To assess the true penetrance of candidate gene alteration and highlight potentially relevant cancer genes, we assessed copy number and mutational data for the 21 genes of interest in a panel consisting of 68 additional in-house NS and smoker LUAD tumors (Fig. 2a, Supplementary Table 4). Mutational status was determined using a custom Illumina capture-based approach while Affymetrix arrays were utilized for copy number determination across the larger LUAD cohort [10]. Overall, mutation frequencies were lower than in the initial WES cohort for the majority of candidates, which may reflect differences in clinical attributes such as smoking status, ethnicity, age or gender as indicated in Fig. 2a.

Oncogenes are typically affected by recurrent mutations clustered at specific genomic hotspots, leading to constitutive protein activation [68]. However, only one of our candidate genes, CELSR2, a member of the flamingo subfamily of non-classic-type cadherins that is thought to have a role in cell-to-cell signaling during nervous system development, demonstrated mutation at the same residue across multiple tumors in our dataset. Thus, we postulated that many of the identified candidates may function as tumor suppressors, as mutations in these genes are commonly dispersed throughout the coding sequence and are inactivating in nature [68]. Indeed, many of the candidates are mutated in conjunction with known driver genes such as *EGFR* and *MET*, suggesting they may cooperate with these oncogenes to promote tumorigenesis. Finally, genes altered at multiple genomic dimensions have been shown to be more likely to be associated with cancer development [69]. For most cancer-related genes, it has been suggested that both genomic copies must be inactivated to cause a phenotypic change in tumorigenesis [70]. Therefore, evidence of “two-hit” alteration can be a useful indicator of a gene’s potential to behave as a tumor-suppressor. We evaluated 12 candidate genes for their frequency of “two-hit” or double alteration across both the TCGA and BCCA cohorts. The well-established tumor-suppressor *TP53* was also evaluated for two-hit frequency as a control. Double alteration included all cases that showed evidence of loss-of-function in both alleles, including homozygous loss (deep deletion), homozygous mutation, or heterozygous mutation combined with loss of the second allele. The most frequent two-hit genes across the TCGA and BCCA cohorts included *SHPRH* and *ASCC3*, with a combined frequency of double alterations of 7% and 6%, respectively (Fig. 2b).

### *SHPRH* as a candidate tumor suppressor gene located within the lung cancer susceptibility locus on chromosome arm 6q

The frequency of double allelic alterations (Fig. 2b) in *SHPRH* and *ASCC3* suggest that they may be candidate tumor suppressor genes in LUAD, whereby their inactivation could contribute to LUAD development and progression. Both genes are located on chromosome 6q, a region of frequent copy number deletion in LUAD [71], with *SHPRH* on chromosome 6q24.3 and *ASCC3* on chromosome 6q16.3. Previous investigation using genome-wide linkage analysis of families with lung cancer revealed that a potential lung cancer susceptibility gene(s) resides within the chromosome 6q23-25 region [9]. Because of its location within this susceptibility locus, *SHPRH* was determined to be the focus of our subsequent follow up to evaluate its candidacy as a tumor suppressor gene in LUAD.

**Fig. 3:**
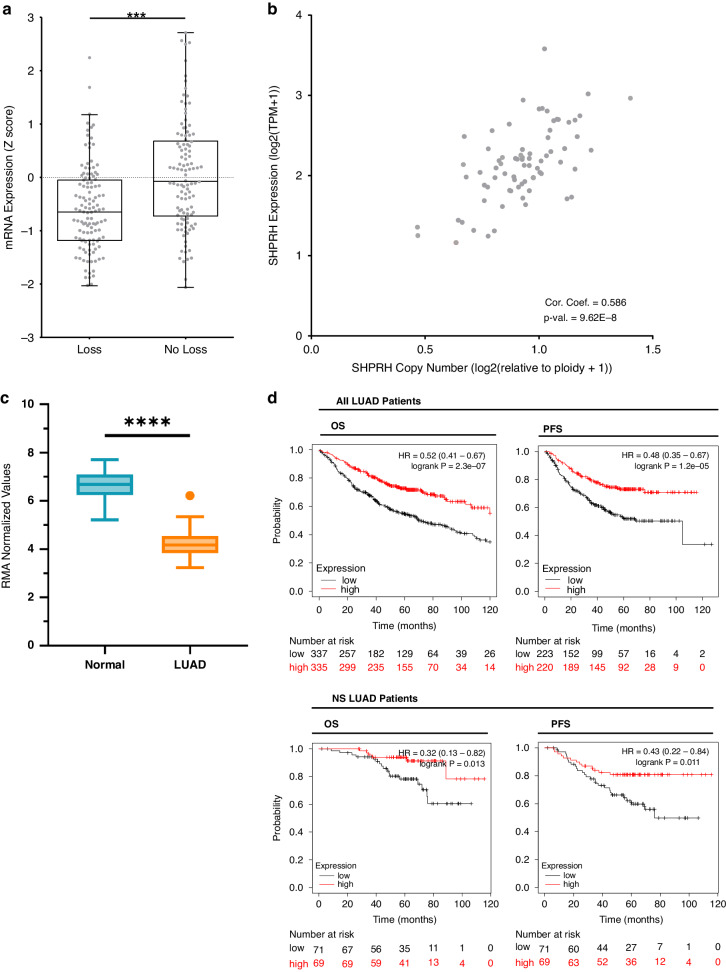
Low *SHPRH* expression is associated with copy number loss and poorer survival outcomes. **a** Box plot showing the association between *SHPRH* mRNA expression and copy number alteration (loss or no loss) in LUAD tumors (*n* = 230). Results from Student’s *t* test are shown, where ***p ≤ 0.001. **b** Scatter plot showing the association between *SHPRH* mRNA expression and copy number in LUAD cell lines (*n* = 74). Pearson correlation coefficient and *p*-value are shown. **c** Box plot showing the level of *SHPRH* expression in LUAD tumors (*N* = 58) compared with normal lung epithelium (*N* = 67). Results from a two-tailed Mann–Whitney U test are shown, where ****p ≤ 0.0001. **d** KM Plots showing overall survival (OS) and progression-free survival (PFS) outcomes in LUAD patients (*n* = 672 for OS, *n* = 528 for PFS) and NS (*n* = 140) LUAD patients. Patients are median split into SHPRH low (black) or high (red) expression. Logrank P-values and Hazard Ratios (HR) from the cox univariate regression model are shown. Data was obtained on 14-Mar-2024.

Examination of copy number alterations in the TCGA LUAD dataset [41] reveals that 52.2% of LUAD tumors (*n* = 230) demonstrate copy number loss of *SHPRH* (Fig. 3a). Furthermore, lower levels of *SHPRH* mRNA are associated with a loss of DNA copy number (Fig. 3a), but does not appear to have an association with the methylation status of *SHPRH* (Supplementary Fig. 2A), suggesting that copy number alterations are the main determinant of *SHPRH* expression in these tumors. Further assessment of LUAD cell lines supports this observation, since *SHPRH* mRNA levels and DNA copy numbers are positively associated (Fig. 3b) [46]. Lastly, expression of *SHPRH* was significantly reduced in LUAD (*N* = 58) in comparison to normal lung epithelium from individuals without lung cancer (*N* = 67), suggesting that *SHPRH* is downregulated/inactivated during cancer development, consistent with a tumor suppressive function (Fig. 3c).

In an expanded analysis of all LUAD datasets available through cBioPortal with available copy number and mutation data, we found that *SHPRH* was mutated or deeply deleted in ~4% (34/980) of tumors, similar in frequency to known LUAD tumor suppressor genes including RB1 (~6%), BRCA2 (~5%), BRCA1 (~3%) and PTEN (~2%) (Supplementary Fig. 2B). In total, 38% (374/980) of the LUADs demonstrated either mutation or copy number loss (deletion or deep deletion).

### *SHPRH* is mutated across different solid cancer types

To determine whether mutation of *SHPRH* is exclusive to LUAD, we next assessed its status across the TCGA PanCancer Atlas datasets. This revealed that *SHPRH* was mutated in 2.4% (251/10443) of all samples profiled, with endometrial carcinoma (11.6%), cutaneous melanoma (5.9%), colorectal adenocarcinoma (4.9%) and stomach adenocarcinoma (4.1%) having the highest mutation rate other than lung cancer (LUAD = 3.9%) (Supplementary Fig. 2C). Furthermore, assessment across cancer types revealed recurrent mutation sites in *SHPRH*, including mutations at G458 (non-sense or frameshift deletions in 8 cases, including LUAD), G587 (missense or frameshift deletions/insertions in 8 cases) and R1365 (R1365C in 5 cases). Indeed, many of the mutations found in LUAD occurred in other cancer types, including those at S93 (3 cases), G183 (2 cases), G458 (8 cases), S725 (2 cases) and R1560 (2 cases) (Supplementary Fig. 2D). Together, this suggest that *SHPRH* may be inactivated in many aggressive solid cancer types in addition to LUAD, with recurrent mutations providing potential evidence of positive selection during tumor evolution.

### Reduced *SHPRH* expression is associated with lower rates of survival in LUAD patients

To explore the clinical implications of SHPRH expression, we assessed survival of LUAD patients in comparison with the levels of *SHPRH* mRNA in their tumors. Patients with LUAD tumors that have low *SHPRH* expression (as determined by a median cutoff for *SHPRH* mRNA expression) have significantly worse overall survival (OS) (*N* = 672) and progression-free survival (PFS) (*N* = 443) than patients with tumors that have high *SHPRH* expression (Fig. 3d, top). This trend also persists when assessing only NS LUAD patients (*N* = 140) (Fig. 3d, bottom). To better represent the proportion of LUAD patients that have a double alteration of *SHPRH*, we further performed survival analysis on a lower quartile cutoff for *SHPRH* mRNA expression and observe a similar association where patients whose tumors have low *SHPRH* expression have poorer disease outcomes, regardless of smoking status (Supplementary Fig. 3A). In contrast, assessment of SCC patients does not reveal an association between *SHPRH* mRNA expression and disease outcomes (Supplementary Fig. 3B). Taken together, this suggests that *SHPRH* may be a candidate tumor suppressor gene with a clinical impact specifically in patients with LUAD tumors.

### Re-expression of SHPRH in lung adenocarcinoma cells with inactivating alterations leads to tumor suppression in vitro and in vivo

**Fig. 4:**
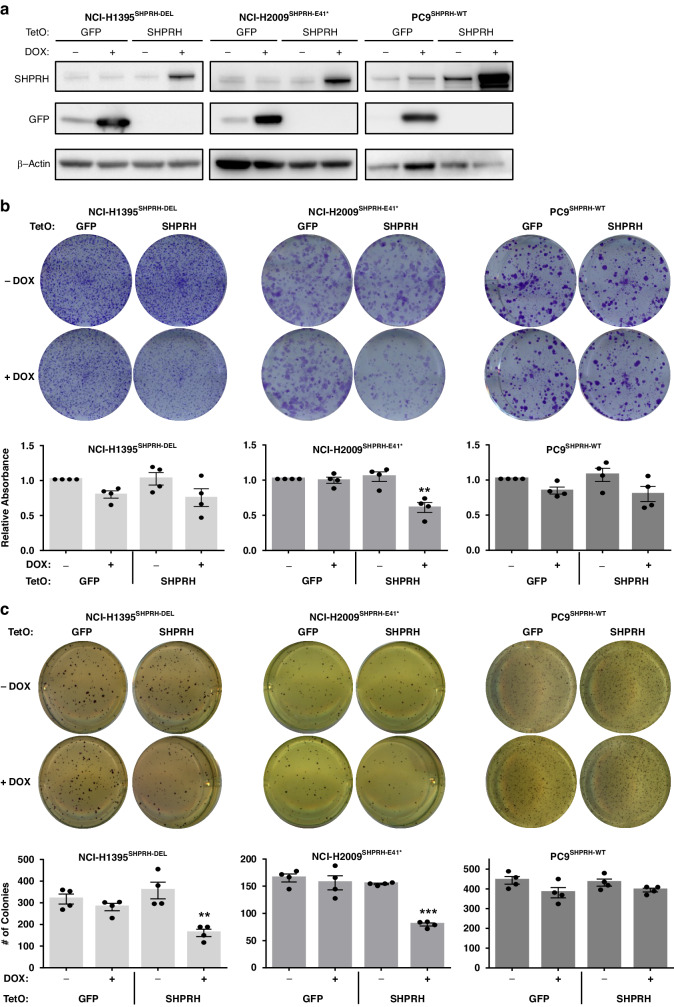
Re-expression of SHPRH specifically reduces colony formation, in vitro, in cells with inactivating alteration of SHPRH. **a** Induction of SHPRH in NCI-H1395^SHPRH-DEL^ (*SHPRH* homozygous deletion), NCI-H2009^SHPRH-E41*^ (*SHPRH* nonsense mutation), and PC9^SHPRH-WT^ (*SHPRH* wildtype) cell lines. GFP or SHPRH was induced by the addition of 100 ng/mL of dox and protein levels were measured by Western blot. **b** Above: Representative images of clonogenic plates showing differences in anchorage-dependent colony growth in TetO GFP and SHPRH cells with or without the addition of 100 ng/mL dox. Below: Quantification of crystal violet staining, calculated relative to GFP no dox condition. Mean ± SEM of *N* = 4 independent replicates is shown. Results from Student’s *t* test are indicated, where *p ≤ 0.05. **c** Above: Representative images of soft agar plates showing differences in anchorage-independent colony formation in TetO GFP and SHPRH cells with or without the addition of 100 ng/mL dox. Below: Quantification of colonies. Mean ± SEM of *N* = 4 independent replicates is shown. Results from Student’s *t* test are indicated, where **p ≤ 0.01, and ***p ≤ 0.001.

We next assessed whether re-expression of SHPRH in LUAD cell lines with biallelic inactivation or mutation of *SHPRH* would decrease their tumorigenic potential. NCI-H1395 has a homozygous deletion of *SHPRH* (NCI-H1395^SHPRH-DEL^), while NCI-H2009 has a nonsense mutation at E41 (NCI-H2009^SHPRH-E41*^). Using a doxycycline(dox)-inducible lentivirus vector system, we transduced these cells to conditionally express SHPRH (TetO SHPRH) or GFP as a control (TetO GFP) (Fig. 4a). This system was also introduced in PC9s, which are wildtype for *SHPRH* (PC9^SHPRH-WT^) (Fig. 4a).

Re-expression of SHPRH in NCI-H1395^SHPRH-DEL^ and NCI-H2009^SHPRH-E41*^ does not alter cell viability after 4 days (Supplementary Fig. 4). However, evaluation of colony growth over longer periods of time shows that re-expression of SHPRH in NCI-H1395^SHPRH-DEL^ significantly reduces the number of anchorage-independent colonies formed in agar, while re-expression of SHPRH in NCI-H2009^SHPRH-E41*^ significantly reduces both anchorage-dependent and -independent colony formation (Fig. 4b, c). In contrast, overexpression of SHPRH in PC9^SHPRH-WT^ does not confer any differences in colony formation (Fig. 4b, c). This suggests that sustained re-expression of SHPRH in cell lines with inactivating alterations of SHPRH can affect their tumorigenic potential, but overexpression in cell lines with functional SHPRH has limited consequences. Re-expression of SHPRH in another homozygous deletion cell line (NCI-H1623^SHPRH-DEL^) and overexpression of SHPRH in a normal lung epithelial cell line (HPL1D^SHPRH-WT^) further supports this observation upon assessment of colony growth (Supplementary Fig. 5), suggesting that the subset of LUADs that evolve to select for SHPRH inactivation are dependent on its suppression for sustained survival.

**Fig. 5:**
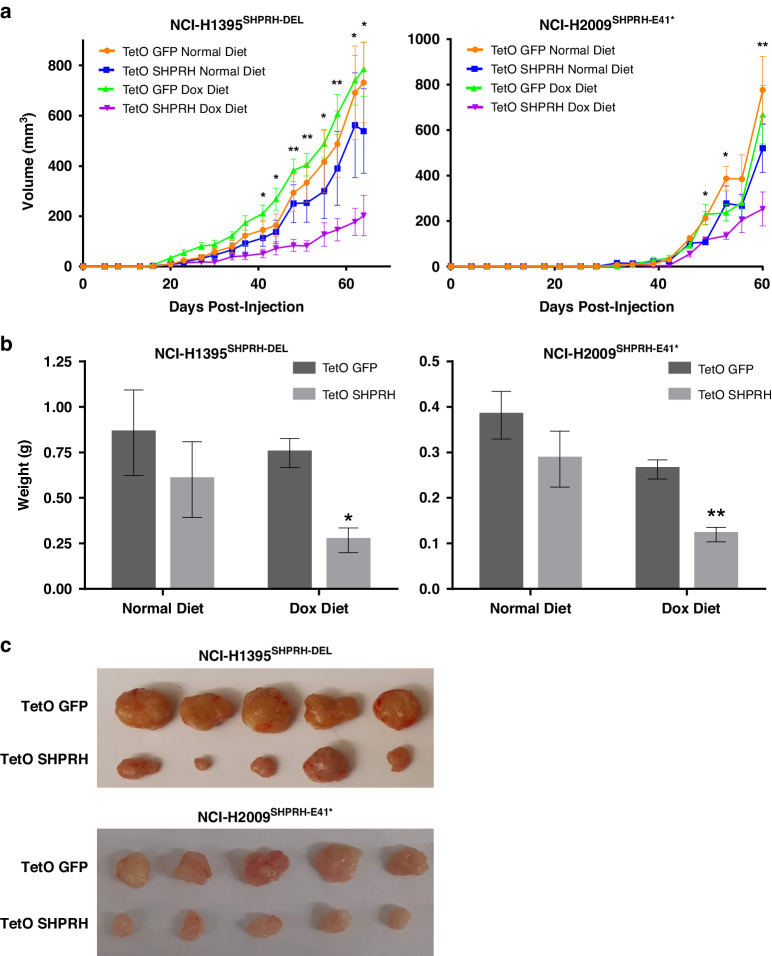
Re-expression of SHPRH reduces tumor formation, in vivo*.* **a** Tumor volume and **b** weight measurements of NCI-H1395^SHPRH-DEL^ and NCI-H2009^SHPRH-E41*^ TetO GFP and SHPRH cells subcutaneously injected in alternating flanks of NRG mice. Mice were kept on either a normal or dox diet. For normal diet, *N* = 6 mice for NCI-H1395 and *N* = 7 mice for NCI-H2009. For dox diet, *N* = 10 mice for NCI-H1395 and *N* = 11 mice for NCI-H2009. Mean ± SEM are shown. Results from Student’s paired *t* test are indicated, where *p ≤ 0.05 and **p ≤ 0.01. **c** Representative images of NCI- H1395^SHPRH-DEL^ and NCI- H2009^SHPRH-E41*^ TetO GFP and SHPRH tumors harvested from mice on a dox diet to demonstrate the smaller size of SHPRH-expressing tumors. See Supplementary Figs. 6 and 7 for images of all tumors and Western blot validation of SHPRH and GFP expression.

Next, we assessed whether re-expression of SHPRH in NCI-H1395^SHPRH-DEL^ and NCI-H2009^SHPRH-E41*^ cells subcutaneously implanted into the flanks of immunocompromised mice kept on a dox diet would affect their ability to form tumors, in vivo. Indeed, TetO SHPRH cells show a significant reduction in tumor growth (Fig. 5a) and size (Fig. 5b, c) in mice kept on a dox diet, whereas TetO SHPRH cells implanted into mice kept on a normal diet do not show a significant difference in comparison to the GFP control.

Lastly, we aimed to confirm that inactivation of *SHPRH* can have a tumor promoting effect by knocking out *SHPRH* with CRISPR in a LUAD cell line with wildtype status (A549) (Supplementary Fig. 8A, B) and performing the same in vivo experiments as described previously. This demonstrated that cells with *SHPRH* knockout had increased growth (Supplementary Fig. 8C) and tumor size (Supplementary Fig. 8D, E) at endpoint compared to their *SHPRH* expressing counterparts, further supporting the role of SHPRH in tumor suppression. *SHPRH* inactivation was confirmed in the tumors collected at endpoint (Supplementary Fig. 8F). Together, this data phenotypically characterizes SHPRH as a tumor suppressor that acts to reduce tumorigenesis in LUAD cells with inactivating alterations of *SHPRH*.

### Re-expression of SHPRH in lung adenocarcinoma cells with inactivating alterations increases cellular fitness in response to DNA damage

**Fig. 6:**
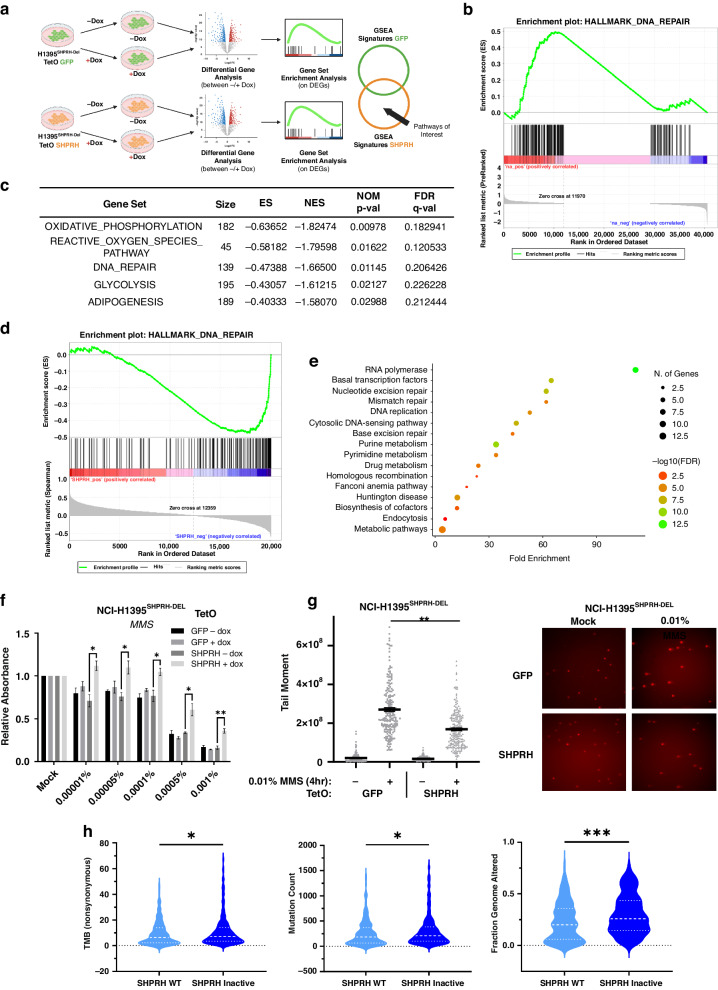
SHPRH expression is negatively correlated with DNA repair-associated genes in LUAD tumors and confers a protective effect to MMS-induced lesions, in vitro. **a** Schematic showing the analysis workflow of RNA-seq data collected from NCI-H1395^SHPRH-DEL^ TetO GFP and SHPRH cells with or without dox. **b** GSEA enrichment plot generated for the Hallmark DNA Repair gene set based on pathways unique to SHPRH expressing NCI-H1395^SHPRH-DEL^ cells. **c** Table outlining the top 5 negatively enriched gene sets within the MSigDB Hallmarks database that are negatively correlated with *SHPRH* expression in LUAD tumors (*N* = 510). Gene set size, enrichment score (ES), normalized enrichment score (NES), nominal *p*-value (NOM p-val), and false discovery rate q-value (FDR q-val) are indicated. **d** GSEA enrichment plot generated for the DNA Repair gene set based on *SHPRH* expression in LUAD tumors. **e** Dot plot showing KEGG pathway analysis of leading-edge genes from the DNA Repair gene set. Dot plot generated using ShinyGO [72, 73]. **f** Quantification of crystal violet staining of clonogenic plates for NCI-H1395^SHPRH-DEL^ TetO GFP and SHPRH cells with or without dox and treated with various concentrations of MMS (or mock-treated control). Calculated relative to the mock-treated condition. Mean ± SEM of *N* = 3 independent replicates are shown. Results from multiple t-tests with correction using the Holm-Sidak method are indicated, where *p ≤ 0.05 and **p ≤ 0.01. **g** *Left*: Quantification of comet tail moment from the neutral comet assay of NCI-H1395^SHPRH-DEL^ TetO GFP and SHPRH cells in dox upon treatment with 0.01% MMS or mock-treated for 4 h. Mean ± SEM of *N* = 3 independent replicates are shown, with 70 events counted per replica. Results from ANOVA are indicated, where **p ≤ 0.01. *Right*: Representative images of the resulting comets. **h** Violin plots outlining the Tumor Mutation Burden (*Left*), Mutation Count (*Center*), and Fraction of Genome Altered (*Right*) in LUAD cases within the TCGA cohort with wildtype or inactivated (mutated or lost) *SHRPH*. Results from one-tailed Mann–Whitney U tests are shown, where *p ≤ 0.05 and ** *p* ≤ 0.01.

To investigate the mechanism by which *SHPRH* may operate as tumor suppressor in LUAD, we next set out to perform RNAseq on the NCI-H1395^SHPRH-DEL^ cells which do not basally express SHPRH. In effort to identify early events which may be driving the tumor suppressive phenotype that is observed in these cells upon re-expression of SHPRH, RNA was collected from TetO GFP and TetO SHPRH cells treated with or without dox for 72 h and then profiled. Differential gene expression analyses (see methods) were performed to compare the dox and no dox conditions for each cell line and GSEA [50, 51] was performed to identify Hallmark [52] gene sets enriched after the addition of dox (Fig. 6a). This analysis revealed that “DNA REPAIR” was the only gene set that was significantly positively enriched in the TetO SHPRH vs TetO GFP cells after the addition of dox (Fig. 6b).

To confirm the clinical relevance of these findings, we next performed GSEA on LUAD patient tumor mRNA expression data (*N* = 510) [49] in order to identify the top gene sets that are negatively correlated with *SHPRH* expression (Fig. 6c), in concordance with *SHPRH*’s proposed function as a tumor suppressor gene. Among the most negatively enriched Hallmark gene sets was “DNA REPAIR” (Fig. 6d), further suggesting a reduction in this gene expression signature may be contributing to LUAD tumorigenesis when *SHPRH* is inactivated. Interestingly, this coincides with SHPRH’s GO annotated functions in DNA repair and cellular response to DNA damage stimulus (Fig. 1c) and its functionally characterized involvement in a mechanism of DNA damage tolerance (DDT) called template switching within literature. Because of this well-established role in DDT, we were interested to determine what specific processes within the DNA REPAIR gene set were affected in tumors with low *SHPRH* expression. We performed KEGG pathway analysis [72, 73] to identify the primary biological actions of the leading edge subset of genes from the DNA REPAIR gene set. This analysis revealed that the top enriched pathways are RNA polymerase, basal transcription factors, nucleotide excision repair (NER), mismatch repair (MMR), and DNA replication (Fig. 6e). Interestingly, SHPRH is known to interact with DNA replication components, transcription factors and RNA polymerase, and along with its paralog HLTF, has recently been demonstrated to have roles in various DNA repair pathways in addition to template switching including MMR and NER, further supporting these findings [74–78].

Based on the above findings, we were interested in exploring whether SHPRH expression affects the tolerance of LUAD cells to DNA damaging lesions. To investigate this in vitro, we re-expressed SHPRH in NCI-H1395^SHPRH-DEL^ and treated the cells with different concentrations of methyl methanesulfonate (MMS), an alkylating agent and carcinogen that methylates the guanine and adenine nucleotides in DNA [79]. MMS was used as a DNA damaging agent in our studies because SHPRH expression has previously been characterized to affect cellular fitness in response to MMS-induced lesions in other cell-based models [80–82]. Therefore, we wanted to see whether similar observations would be had in the context of LUAD. Furthermore, this was assessed in NCI-H1395^SHPRH-DEL^ because it was observed that SHPRH re-expression alone does not affect anchorage-dependent colony formation (Fig. 4b) and therefore provides a more accurate comparison of cell survival in the presence of a genotoxic agent such as MMS.

Using clonogenic survival assays to assess for alterations in cell survival, we observed that re-expression of SHPRH in NCI-H1395^SHPRH-DEL^ cells reduces their sensitivity to MMS compared to control conditions (Fig. 6f). In addition, assessment of these cells upon MMS treatment using the neutral comet assay revealed that they have a reduction in their overall tail moment compared to GFP-expressing cells (Fig. 6g), suggesting that the SHPRH-expressing cells have a reduced amount of double stranded breaks.

Lastly, to determine whether evidence of increased DNA damage is associated with SHPRH inactivation in LUAD patients we separated tumors based on SHPRH inactivation status (mutation or deletion vs wildtype) and compared the degree of Tumor Mutational Burden (TMB), Mutational Count, and Fraction Genome Altered using sequence and clinical data from the LUAD TCGA PanCancer dataset [49]. This revealed that LUADs with SHPRH inactivation had significantly more TMB, Mutational Count and Fraction Genome Altered, consistent with a role of this gene in mediating processes related to DNA repair (Fig. 6h). Additionally, two-hit alterations of SHPRH had significantly more TMB and Mutational Count compared to one-hit alterations (Supplementary Fig. 9). Taken together, these experimental and clinical findings suggest that SHPRH expression may have a role in the tolerance of DNA damage during LUAD tumorigenesis.

### *SHPRH* inactivation increases sensitivity to therapeutic agents

**Fig. 7:**
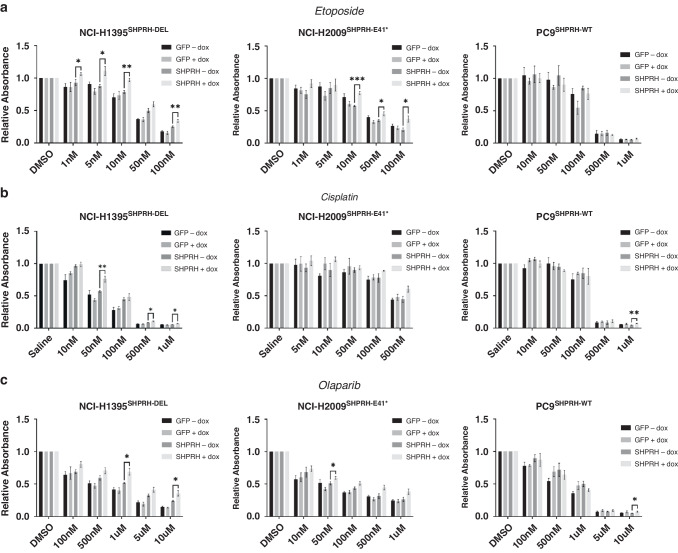
SHPRH expression affects colony formation in response to certain chemotherapeutics and targeted therapies, in vitro*.* Quantification of crystal violet staining of clonogenic plates for NCI-H1395^SHPRH-DEL^, NCI-H2009^SHPRH-E41*^, and PC9^SHPRH-WT^ TetO GFP and SHPRH cells with or without dox and treated with various concentrations of (**a**) etoposide (or DMSO control), (**b**) cisplatin (or saline control), and (**c**) olaparib (or DMSO control). Absorbance are calculated relative to the saline or DMSO control conditions. Mean ± SEM of *N* = 3 independent replicates are shown. Results from multiple t-tests without correction are indicated, where *p < 0.05, **p ≤ 0.01, and ***p ≤ 0.001.

Finally, *SHPRH* status may also serve to highlight targetable vulnerabilities and predict response to therapeutic agents in LUAD. Given its proposed role in mediating DNA damage response, SHPRH expression may affect tolerance to various types of DNA lesions targeted by numerous chemotherapeutic and inhibitors currently employed in the clinic. Indeed, there is reported evidence to suggest that SHPRH expression may predict response to various therapeutic agents across several cell types [83–85]. Therefore, to assess whether *SHPRH* expression can help mediate the tolerance of LUAD cells to DNA damaging chemotherapeutics (etoposide and cisplatin) and PARP inhibitors (olaparib), we performed clonogenic survival assays on NCI-H1395^SHPRH-DEL^, NCI-H2009^SHPRH-E41^ and PC9^SHPRH-WT^ TetO GFP and TetO SHPRH cells (Fig. 7). Etoposide and cisplatin are presently used as first line adjuvant or neoadjuvant therapies to treat NSCLC, while olaparib is currently not approved for use in lung cancer but has been explored as a possible therapeutic for chemo-sensitive NSCLC tumor control in clinical trials [86]. Interestingly, *SHPRH* re-expression appears to significantly confer a protective effect in response to etoposide treatment across multiple concentrations in cell lines with inactive *SHPRH* (H1395 and H2009) (Fig. 7a), with a moderate effect seen upon cisplatin and olaparib treatment (Fig. 7b, c). In contrast, overexpression of *SHPRH* in *SHPRH* wildtype PC9 cells demonstrated no difference at effective concentration for these agents. Together, this suggests that the absence of functional SHPRH in LUAD may provide a vulnerability to clinical therapeutics that target DNA damage response.

## Discussion

The discovery and identification of cancer-associated genes has been transformative for our understanding of LUAD. In recent decades, the improvement and diversification of sequencing platforms has allowed for a greater emphasis to be placed on characterizing the genomic landscape of lung cancer to direct molecular testing, identify therapeutic targets, and inform cancer care via the development of personalized therapies for patients [87]. However, while our understanding and targeting of the specific oncogenic alterations driving lung cancer has progressed significantly, considerably less is known about the tumor suppressive landscape of LUAD outside of the limited number of well-characterized tumor suppressors such as RB, TP53, P16, and LKB1. By focusing on integrative genomic analyses using WES of 15 NS LUAD tumors supplemented with custom capture and copy number analyses of an expanded cohort of 68 in-house LUAD tumors and validated with external LUAD datasets, we were able to identify several novel tumor suppressor candidates in LUAD with minimal confounding from passenger gene alterations that can be caused by smoking.

While the mutations of a gene such as *SHPRH* appear functionally relevant for tumorigenesis, the absence of hotspot mutations in individual cancer types and overall size of this gene could prevent it from reaching statistical relevance in commonly used computational pipelines that assess mutational significance [88], some of which were built on specific observations that may not be transferable to all cancer types or study conditions [89]. Thus, while these algorithms are helpful for identification of cancer drivers, they may have a degree of false negatives, underscoring the relevance of using various approaches for discovery of cancer associated genes. The workflow described here uses a unique filtering method, which identifies candidates based on mutations that are predicted to have a significant functional impact. Indeed, genes that have been well-documented in other cancers yet have not been given much clinical interest in lung cancer are demonstrated in our data. This may be due to the low penetrance of these mutations and relatively small number of LUADs from NSs subjected to DNA sequencing to date. Further evaluation may continue to lead to better understanding of lung cancer biology and the application of novel treatment strategies that are being assessed in other cancer types for a subset of patients with relevant alterations in these genes.

Because of the predicted functional impact in our tumor population, the frequency of double allelic disruptions in the expanded datasets, and its chromosomal location in a major lung cancer susceptibility region [9], we propose that *SHPRH* is a candidate tumor suppressor gene in LUAD. Across several cancer types, there has been an observed loss of heterozygosity of chromosome 6q24 [90], further suggesting that *SHPRH* – located at 6q24.3 – may have a tumor suppressive role in cancer. In ovarian cancer, germline mutation of *SHPRH* is associated with a moderate-to-high risk of developing ovarian cancer [91], while in colon cancer, SHPRH has been shown to inhibit Wnt signaling [92]. This suggests that its inactivation may also be involved in tumor initiation or progression. Furthermore, recent studies of a novel protein encoded by a circular RNA form of SHPRH (circ-SHPRH) has identified it as having a tumor suppressive function in glioblastoma [93]. However, while it was suggested that this protein operated by protecting the full-length SHPRH protein from degradation, no further investigation was done on the function of full-length SHPRH itself in glioblastoma [93]. Similarly, while there has been a recent emergence of evidence suggesting that circ-SHPRH operates as a tumor suppressor gene in a multitude of other cancers – where its downregulation in turn promotes cancer development and progression by affecting a variety of oncogenic processes – the evaluation of its effect on full-length SHPRH in these cancers has yet to be conducted [94]. As such, despite the overwhelming evidence suggesting that full-length SHPRH may be a tumor suppressor in multiple cancer types, the functional characterization of it as a tumor suppressor gene has yet to be exhibited.

In this study, we showed that low SHPRH expression is associated with poorer overall survival outcomes in LUAD patients, regardless of smoking status. Furthermore, we were able to phenotypically characterize SHPRH as a tumor suppressor gene by demonstrating that SHPRH re-expression in LUAD cell lines with inactivating alterations of SHPRH reduces their tumorigenic potential, in vitro and in vivo. Interestingly, we only observed this in cell lines with biallelic inactivation or mutation of SHPRH, while overexpression of SHPRH in cell lines with wildtype SHPRH had no impact on their tumorigenic potential, suggesting that its inactivation plays an integral role in the tumorigenicity of these cells. Furthermore, GSEA of LUAD tumors revealed an enrichment for DNA repair-associated genes that is negatively correlated with *SHPRH* expression and a preliminary investigation into the mechanistic effect that SHPRH expression may have on the tolerance of DNA damage suggests it may be protective against MMS-induced lesions and therapeutic agents that induce DNA damage. However, further investigation is needed to better understand the role of SHPRH in LUAD development, progression, and outcome.

Chromosome 6q is a region of frequent copy number deletion [71] and has been suggested to contain tumor suppressor genes [95–97]. Previous studies using family-based linkage analysis have also found that a major lung cancer susceptibility locus located at chromosome 6q23-25 is frequently deleted in families with lung cancer [9]. Specifically, it was noted that a lung cancer susceptibility gene(s) is co-inherited with a genetic marker in the chromosome 6q23-25 region in an estimated 67% of families, which increased with more affected relatives. Furthermore, carriers of alterations in this susceptibility region experience a significantly enhanced risk for developing lung cancer than non-carriers, which was further augmented upon the introduction of smoking [9, 98]. This suggests that a tumor suppressor gene or several tumor suppressor genes may be responsible for these findings and that the biological function of the susceptibility gene(s) may work alongside the carcinogenic effects of smoking to promote lung cancer development. While subsequent fine mapping studies have identified several genes as being candidate susceptibility markers in this locus, including *SASH1, RGS17*, and *EYA4* [99–101], limited progress has been made to comprehensively characterize these genes as independent or cooperative tumor suppressor genes with a hereditary and prognostic role in lung cancer development. Because *SHPRH* resides within this lung cancer susceptibility region at chromosome 6q24.3 and is an appealing candidate for a tumor suppressor gene with an implicated association with LUAD, further investigation into its role in lung cancer risk and development would be of interest.

SHPRH is an E3 ubiquitin ligase involved in mediating template switching, a mechanism of DNA damage tolerance to single stranded lesions. Template switching helps maintain the integrity of the genome by using homologous recombination-based machinery during replication to bypass such lesions in the replication fork, which would otherwise cause for the replication fork to stall and become prone to breakage if left unresolved [74, 102]. Indeed, our investigations suggest that SHPRH expression in LUAD cells may confer a protective effect against cellular toxicity and double-stranded breaks in response to MMS-induced lesions; however, further investigation of its implication for the cell’s overall genomic stability and an understanding of the mechanism by which SHPRH operates in the face of DNA damage – MMS-induced and otherwise – is needed. Because of its role in DNA damage tolerance, SHPRH may work to counteract the effects of lung cancer risk factors, such as smoking, environmental factors, and intrinsic cellular DNA damage, in order to help prevent lung cancer development and progression. In support of this, we show that *SHPRH* inactivation is associated with increased mutations and genomic instability in LUAD, confirming a recent report demonstrating a similar association across TCGA datasets [77]. In addition, investigating the cooperative effect of *SHPRH* inactivation with other known risk factors or genetic drivers of lung cancer – such as *EGFR* or *KRAS* mutations – may provide further understanding of its role in LUAD biology, such that the inactivation of *SHPRH* may make cells more permissive to undergo malignant transformation in the presence of these cooperating partners. Finally, we show that SHPRH may also serve to be a predictor of response to therapeutic agents, whereby inactivation of *SHPRH* is associated with better efficacy of drug therapies that operate by inducing overwhelming amounts of DNA damage to the cancer cells, providing a potential strategy for treatment of LUAD patients.

## Conclusions

In conclusion, our study has identified several novel candidate genetic drivers that have a predicted functional consequence in LUAD. One of the genes, *SHPRH*, was of particular interest due to its frequency of double allelic alterations and location in a lung cancer susceptibility region. Through clinical and biological investigations, we were able to characterize SHPRH as a tumor suppressor gene in LUAD whose expression is associated with a more favorable prognosis. However, further understanding of its role in LUAD tumorigenesis is required. Because of its function as an E3 ubiquitin ligase, the identification of SHPRH-interacting proteins paired with an analysis of the transcriptome in SHPRH-expressing LUAD may help guide our understanding of the influence that SHPRH expression has in mediating LUAD development and progression. Similarly, explorations into its reported role in cellular functions such as DNA repair, cellular response to DNA damage stimulus, and others identified by the GSEA results of LUAD cell lines and tumors may yield a greater appreciation of SHPRH’s role in response to extrinsic and intrinsic factors affecting LUAD tumorigenesis. In doing so, SHPRH may become an important genetic marker to identify at-risk individuals for lung cancer and to highlight a targetable vulnerability in these patients.

### Supplementary information

Supplemental Figures and Legends

Supplemental Table 1

Supplemental Table 2

Supplemental Table 3

Supplemental Table 4

## Data Availability

The data from this study have been submitted to NCBI BioProject under BioProject number PRJNA985279.

## References

[1] Sung H, Ferlay J, Siegel RL, Laversanne M, Soerjomataram I, Jemal A, et al. Global Cancer Statistics 2020: GLOBOCAN Estimates of Incidence and Mortality Worldwide for 36 Cancers in 185 Countries. CA Cancer J Clin. 2021;71:209–49. 33538338 10.3322/caac.21660

[2] Canadian Cancer Statistics Advisory Committee. Canadian Cancer Statistics: A 2020 special report on lung cancer [PDF]. Toronto, ON: Canadian Cancer Society; 2020. Available from: cancer.ca/Canadian-Cancer-Statistics-2020-EN.

[3] Canadian Cancer Statistics Advisory Committee in collaboration with the Canadian Cancer Society. Canadian Cancer Statistics 2021 [PDF]: Canadian Cancer Society; 2021. Available from: cancer.ca/Canadian-Cancer-Statistics-2021-EN.

[4] Lemjabbar-Alaoui H, Hassan OU, Yang YW, Buchanan P. Lung cancer: Biology and treatment options. Biochim Biophys Acta. 2015;1856:189–210. 26297204 10.1016/j.bbcan.2015.08.002 PMC4663145

[5] Yuan M, Huang L-L, Chen J-H, Wu J, Xu Q. The emerging treatment landscape of targeted therapy in non-small-cell lung cancer. Signal Transduct Target Ther. 2019;4:61. 31871778 10.1038/s41392-019-0099-9 PMC6914774

[6] Couraud S, Zalcman G, Milleron B, Morin F, Souquet P-J. Lung cancer in never smokers-a review. Eur J Cancer. 2012;48:1299–311. 22464348 10.1016/j.ejca.2012.03.007

[7] Lawrence MS, Stojanov P, Mermel CH, Robinson JT, Garraway LA, Golub TR, et al. Discovery and saturation analysis of cancer genes across 21 tumour types. Nature. 2014;505:495–501. 24390350 10.1038/nature12912 PMC4048962

[8] Pao W, Miller V, Zakowski M, Doherty J, Politi K, Sarkaria I, et al. EGF receptor gene mutations are common in lung cancers from “never smokers” and are associated with sensitivity of tumors to gefitinib and erlotinib. Proc Natl Acad Sci USA. 2004;101:13306–11. 15329413 10.1073/pnas.0405220101 PMC516528

[9] Bailey-Wilson JE, Amos CI, Pinney SM, Petersen GM, de Andrade M, Wiest JS, et al. A major lung cancer susceptibility locus maps to chromosome 6q23-25. Am J Hum Genet. 2004;75:460–74. 15272417 10.1086/423857 PMC1182024

[10] O’Farrell H, Harbourne B, Kurlawala Z, Inoue Y, Nagelberg AL, Martinez VD, et al. Integrative Genomic Analyses Identifies GGA2 as a Cooperative Driver of EGFR-Mediated Lung Tumorigenesis. J Thorac Oncol. 2019;14:656–71. 30578931 10.1016/j.jtho.2018.12.004 PMC6440869

[11] Le Gallo M, O’Hara AJ, Rudd ML, Urick ME, Hansen NF, O’Neil NJ, et al. Exome sequencing of serous endometrial tumors identifies recurrent somatic mutations in chromatin-remodeling and ubiquitin ligase complex genes. Nat Genet. 2012;44:1310–5. 23104009 10.1038/ng.2455 PMC3515204

[12] Wei X, Walia V, Lin JC, Teer JK, Prickett TD, Gartner J, et al. Exome sequencing identifies GRIN2A as frequently mutated in melanoma. Nat Genet. 2011;43:442–6. 21499247 10.1038/ng.810 PMC3161250

[13] Li H. A statistical framework for SNP calling, mutation discovery, association mapping and population genetical parameter estimation from sequencing data. Bioinformatics. 2011;27:2987–93. 21903627 10.1093/bioinformatics/btr509 PMC3198575

[14] Koboldt DC, Chen K, Wylie T, Larson DE, McLellan MD, Mardis ER, et al. VarScan: variant detection in massively parallel sequencing of individual and pooled samples. Bioinformatics. 2009;25:2283–5. 19542151 10.1093/bioinformatics/btp373 PMC2734323

[15] Garrison E, Marth G. Haplotype-based variant detection from short-read sequencing. arXiv. 2012. https://arxiv.org/abs/1207.3907.

[16] Danecek P, Bonfield JK, Liddle J, Marshall J, Ohan V, Pollard MO, et al. Twelve years of SAMtools and BCFtools. Gigascience. 2021;10:giab008. 33590861 10.1093/gigascience/giab008 PMC7931819

[17] Wang K, Li M, Hakonarson H. ANNOVAR: functional annotation of genetic variants from high-throughput sequencing data. Nucleic Acids Res. 2010;38:e164. 20601685 10.1093/nar/gkq603 PMC2938201

[18] Chen S, Huang T, Wen T, Li H, Xu M, Gu J. MutScan: fast detection and visualization of target mutations by scanning FASTQ data. BMC Bioinforma. 2018;19:16. 10.1186/s12859-018-2024-6 PMC5778627 29357822

[19] Mayakonda A, Lin D-C, Assenov Y, Plass C, Koeffler HP. Maftools: efficient and comprehensive analysis of somatic variants in cancer. Genome Res. 2018;28:1747–56. 30341162 10.1101/gr.239244.118 PMC6211645

[20] Wickham H, François R, Henry L, Müller K. dplyr:A Grammar of Data Manipulation. 1.0.5 ed. R package. 2021.

[21] Li H, Handsaker B, Wysoker A, Fennell T, Ruan J, Homer N, et al. The Sequence Alignment/Map format and SAMtools. Bioinformatics. 2009;25:2078–9. 19505943 10.1093/bioinformatics/btp352 PMC2723002

[22] Sherry ST, Ward MH, Kholodov M, Baker J, Phan L, Smigielski EM, et al. dbSNP: the NCBI database of genetic variation. Nucleic Acids Res. 2001;29:308–11. 11125122 10.1093/nar/29.1.308 PMC29783

[23] Genomes Project C, Auton A, Brooks LD, Durbin RM, Garrison EP, Kang HM, et al. A global reference for human genetic variation. Nature. 2015;526:68–74. 26432245 10.1038/nature15393 PMC4750478

[24] Tennessen JA, Bigham AW, O’Connor TD, Fu W, Kenny EE, Gravel S, et al. Evolution and functional impact of rare coding variation from deep sequencing of human exomes. Science. 2012;337:64–9. 22604720 10.1126/science.1219240 PMC3708544

[25] Lek M, Karczewski KJ, Minikel EV, Samocha KE, Banks E, Fennell T, et al. Analysis of protein-coding genetic variation in 60,706 humans. Nature. 2016;536:285–91. 27535533 10.1038/nature19057 PMC5018207

[26] O’Leary NA, Wright MW, Brister JR, Ciufo S, Haddad D, McVeigh R, et al. Reference sequence (RefSeq) database at NCBI: current status, taxonomic expansion, and functional annotation. Nucleic Acids Res. 2016;44:D733–45. 26553804 10.1093/nar/gkv1189 PMC4702849

[27] Xiong HY, Alipanahi B, Lee LJ, Bretschneider H, Merico D, Yuen RKC, et al. RNA splicing. The human splicing code reveals new insights into the genetic determinants of disease. Science. 2015;347:1254806. 25525159 10.1126/science.1254806 PMC4362528

[28] Imielinski M, Berger AH, Hammerman PS, Hernandez B, Pugh TJ, Hodis E, et al. Mapping the hallmarks of lung adenocarcinoma with massively parallel sequencing. Cell. 2012;150:1107–20. 22980975 10.1016/j.cell.2012.08.029 PMC3557932

[29] Ng PC, Henikoff S. SIFT: Predicting amino acid changes that affect protein function. Nucleic Acids Res. 2003;31:3812–4. 12824425 10.1093/nar/gkg509 PMC168916

[30] Chun S, Fay JC. Identification of deleterious mutations within three human genomes. Genome Res. 2009;19:1553–61. 19602639 10.1101/gr.092619.109 PMC2752137

[31] Schwarz JM, Cooper DN, Schuelke M, Seelow D. MutationTaster2: mutation prediction for the deep-sequencing age. Nat Methods. 2014;11:361–2. 24681721 10.1038/nmeth.2890

[32] Reva B, Antipin Y, Sander C. Predicting the functional impact of protein mutations: application to cancer genomics. Nucleic Acids Res. 2011;39:e118. 21727090 10.1093/nar/gkr407 PMC3177186

[33] Shihab HA, Gough J, Cooper DN, Day INM, Gaunt TR. Predicting the functional consequences of cancer-associated amino acid substitutions. Bioinformatics. 2013;29:1504–10. 23620363 10.1093/bioinformatics/btt182 PMC3673218

[34] Shihab HA, Gough J, Cooper DN, Stenson PD, Barker GLA, Edwards KJ, et al. Predicting the functional, molecular, and phenotypic consequences of amino acid substitutions using hidden Markov models. Hum Mutat. 2013;34:57–65. 23033316 10.1002/humu.22225 PMC3558800

[35] Shihab HA, Gough J, Mort M, Cooper DN, Day INM, Gaunt TR. Ranking non-synonymous single nucleotide polymorphisms based on disease concepts. Hum Genomics. 2014;8:11. 24980617 10.1186/1479-7364-8-11 PMC4083756

[36] Dong C, Wei P, Jian X, Gibbs R, Boerwinkle E, Wang K, et al. Comparison and integration of deleteriousness prediction methods for nonsynonymous SNVs in whole exome sequencing studies. Hum Mol Genet. 2015;24:2125–37. 25552646 10.1093/hmg/ddu733 PMC4375422

[37] Thu KL, Vucic EA, Chari R, Zhang W, Lockwood WW, English JC, et al. Lung adenocarcinoma of never smokers and smokers harbor differential regions of genetic alteration and exhibit different levels of genomic instability. PLoS One. 2012;7:e33003. 22412972 10.1371/journal.pone.0033003 PMC3296775

[38] Mermel CH, Schumacher SE, Hill B, Meyerson ML, Beroukhim R, Getz G. GISTIC2.0 facilitates sensitive and confident localization of the targets of focal somatic copy-number alteration in human cancers. Genome Biol. 2011;12:R41. 21527027 10.1186/gb-2011-12-4-r41 PMC3218867

[39] Beroukhim R, Mermel CH, Porter D, Wei G, Raychaudhuri S, Donovan J, et al. The landscape of somatic copy-number alteration across human cancers. Nature. 2010;463:899–905. 20164920 10.1038/nature08822 PMC2826709

[40] Lockwood WW, Wilson IM, Coe BP, Chari R, Pikor LA, Thu KL, et al. Divergent genomic and epigenomic landscapes of lung cancer subtypes underscore the selection of different oncogenic pathways during tumor development. PLoS One. 2012;7:e37775. 22629454 10.1371/journal.pone.0037775 PMC3357406

[41] Cancer Genome Atlas Research N. Comprehensive molecular profiling of lung adenocarcinoma. Nature. 2014;511:543–50. 25079552 10.1038/nature13385 PMC4231481

[42] Cerami E, Gao J, Dogrusoz U, Gross BE, Sumer SO, Aksoy BA, et al. The cBio cancer genomics portal: an open platform for exploring multidimensional cancer genomics data. Cancer Discov. 2012;2:401–4. 22588877 10.1158/2159-8290.CD-12-0095 PMC3956037

[43] Gao J, Aksoy BA, Dogrusoz U, Dresdner G, Gross B, Sumer SO, et al. Integrative analysis of complex cancer genomics and clinical profiles using the cBioPortal. Sci Signal. 2013;6:pl1. 23550210 10.1126/scisignal.2004088 PMC4160307

[44] Chari R, Lonergan KM, Ng RT, MacAulay C, Lam WL, Lam S. Effect of active smoking on the human bronchial epithelium transcriptome. BMC Genomics. 2007;8:297. 17727719 10.1186/1471-2164-8-297 PMC2001199

[45] Lockwood WW, Chari R, Coe BP, Thu KL, Garnis C, Malloff CA, et al. Integrative genomic analyses identify BRF2 as a novel lineage-specific oncogene in lung squamous cell carcinoma. PLoS Med. 2010;7:e1000315. 20668658 10.1371/journal.pmed.1000315 PMC2910599

[46] DepMap B DepMap 19Q3 Public. Figshare. 2019. https://depmap.org/portal/data_page/?tab=overview#how-to-cite.

[47] Ghandi M, Huang FW, Jané-Valbuena J, Kryukov GV, Lo CC, McDonald ER 3rd, et al. Next-generation characterization of the Cancer Cell Line Encyclopedia. Nature 2019;569:503–8. 31068700 10.1038/s41586-019-1186-3 PMC6697103

[48] Győrffy B, Surowiak P, Budczies J, Lánczky A. Online survival analysis software to assess the prognostic value of biomarkers using transcriptomic data in non-small-cell lung cancer. PLoS ONE. 2013;8:e82241. 24367507 10.1371/journal.pone.0082241 PMC3867325

[49] Weinstein JN, Collisson EA, Mills GB, Shaw KR, Ozenberger BA, Ellrott K, et al. The Cancer Genome Atlas Pan-Cancer analysis project. Nat Genet. 2013;45:1113–20. 24071849 10.1038/ng.2764 PMC3919969

[50] Subramanian A, Tamayo P, Mootha VK, Mukherjee S, Ebert BL, Gillette MA, et al. Gene set enrichment analysis: a knowledge-based approach for interpreting genome-wide expression profiles. Proc Natl Acad Sci USA. 2005;102:15545–50. 16199517 10.1073/pnas.0506580102 PMC1239896

[51] Mootha VK, Lindgren CM, Eriksson KF, Subramanian A, Sihag S, Lehar J, et al. PGC-1alpha-responsive genes involved in oxidative phosphorylation are coordinately downregulated in human diabetes. Nat Genet. 2003;34:267–73. 12808457 10.1038/ng1180

[52] Liberzon A, Subramanian A, Pinchback R, Thorvaldsdóttir H, Tamayo P, Mesirov JP. Molecular signatures database (MSigDB) 3.0. Bioinformatics. 2011;27:1739–40. 21546393 10.1093/bioinformatics/btr260 PMC3106198

[53] Uphoff CC, Drexler HG. Detecting mycoplasma contamination in cell cultures by polymerase chain reaction. Methods Mol Biol. 2011;731:93–103. 21516400 10.1007/978-1-61779-080-5_8

[54] Meerbrey KL, Hu G, Kessler JD, Roarty K, Li MZ, Fang JE, et al. The pINDUCER lentiviral toolkit for inducible RNA interference in vitro and in vivo. Proc Natl Acad Sci USA. 2011;108:3665–70. 21307310 10.1073/pnas.1019736108 PMC3048138

[55] Shalem O, Sanjana NE, Hartenian E, Shi X, Scott DA, Mikkelsen TS, et al. Genome-Scale CRISPR-Cas9 Knockout Screening in Human Cells. Science. 2014;343:84–7. 24336571 10.1126/science.1247005 PMC4089965

[56] Sanjana NE, Shalem O, Zhang F. Improved vectors and genome-wide libraries for CRISPR screening. Nat Methods. 2014;11:783–4. 25075903 10.1038/nmeth.3047 PMC4486245

[57] Schindelin J, Arganda-Carreras I, Frise E, Kaynig V, Longair M, Pietzsch T, et al. Fiji: an open-source platform for biological-image analysis. Nat Methods. 2012;9:676–82. 22743772 10.1038/nmeth.2019 PMC3855844

[58] Mathew V, Pauleau AL, Steffen N, Bergner A, Becker PB, Erhardt S. The histone-fold protein CHRAC14 influences chromatin composition in response to DNA damage. Cell Rep. 2014;7:321–30. 24703848 10.1016/j.celrep.2014.03.008

[59] Babraham Bioinformatics. FastQC 2023. Available from https://www.bioinformatics.babraham.ac.uk/projects/fastqc/.

[60] Martin M. Cutadapt removes adapter sequences from high-throughput sequencing reads. EMBnetjournal. 2011;17:10.

[61] Patro R, Duggal G, Love MI, Irizarry RA, Kingsford C. Salmon provides fast and bias-aware quantification of transcript expression. Nat Methods. 2017;14:417–9. 28263959 10.1038/nmeth.4197 PMC5600148

[62] RCore Team. R: A language and environment for statistical computing. R Foundation for Statistical Computing; 2024. https://www.r-project.org/.

[63] Soneson C, Love M, Robinson M. Differential analyses for RNA-seq: transcript-level estimates improve gene-level inferences [version 2; peer review: 2 approved]. F1000Research. 2016;4:1521. 10.12688/f1000research.7563.2 PMC4712774 26925227

[64] Mertz JA, Conery AR, Bryant BM, Sandy P, Balasubramanian S, Mele DA, et al. Targeting MYC dependence in cancer by inhibiting BET bromodomains. Proc Natl Acad Sci USA. 2011;108:16669–74. 21949397 10.1073/pnas.1108190108 PMC3189078

[65] Lockwood WW, Zejnullahu K, Bradner JE, Varmus H. Sensitivity of human lung adenocarcinoma cell lines to targeted inhibition of BET epigenetic signaling proteins. Proc Natl Acad Sci USA. 2012;109:19408–13. 23129625 10.1073/pnas.1216363109 PMC3511085

[66] Tate JG, Bamford S, Jubb HC, Sondka Z, Beare DM, Bindal N, et al. COSMIC: the catalogue of somatic mutations in cancer. Nucleic Acids Res. 2019;47:D941–D7. 30371878 10.1093/nar/gky1015 PMC6323903

[67] Soh J, Okumura N, Lockwood WW, Yamamoto H, Shigematsu H, Zhang W, et al. Oncogene mutations, copy number gains and mutant allele specific imbalance (MASI) frequently occur together in tumor cells. Plos One. 2009;4:e7464. 19826477 10.1371/journal.pone.0007464 PMC2757721

[68] Vogelstein B, Papadopoulos N, Velculescu VE, Zhou S, Diaz LA Jr, Kinzler KW. Cancer genome landscapes. Science. 2013;339:1546–58. 23539594 10.1126/science.1235122 PMC3749880

[69] Chari R, Coe BP, Vucic EA, Lockwood WW, Lam WL. An integrative multi-dimensional genetic and epigenetic strategy to identify aberrant genes and pathways in cancer. BMC Syst Biol. 2010;4:67. 20478067 10.1186/1752-0509-4-67 PMC2880289

[70] Knudson AG Jr. Mutation and cancer: statistical study of retinoblastoma. Proc Natl Acad Sci USA. 1971;68:820–3. 5279523 10.1073/pnas.68.4.820 PMC389051

[71] Weir BA, Woo MS, Getz G, Perner S, Ding L, Beroukhim R, et al. Characterizing the cancer genome in lung adenocarcinoma. Nature. 2007;450:893–8. 17982442 10.1038/nature06358 PMC2538683

[72] Ge SX, Jung D, Yao R. ShinyGO: a graphical gene-set enrichment tool for animals and plants. Bioinformatics. 2020;36:2628–9. 31882993 10.1093/bioinformatics/btz931 PMC7178415

[73] Kanehisa M, Furumichi M, Sato Y, Ishiguro-Watanabe M, Tanabe M. KEGG: integrating viruses and cellular organisms. Nucleic Acids Res. 2021;49:D545–d51. 33125081 10.1093/nar/gkaa970 PMC7779016

[74] Seelinger M, Sogaard CK, Otterlei M. The Human RAD5 Homologs, HLTF and SHPRH, Have Separate Functions in DNA Damage Tolerance Dependent on The DNA Lesion Type. Biomolecules. 2020;10:463. 32192191 10.3390/biom10030463 PMC7175315

[75] Miller AK, Mao G, Knicely BG, Daniels HG, Rahal C, Putnam CD, et al. Rad5 and Its Human Homologs, HLTF and SHPRH, Are Novel Interactors of Mismatch Repair. Front Cell Dev Biol. 2022;10:843121. 35784486 10.3389/fcell.2022.843121 PMC9243396

[76] Lee D, An J, Park Y-U, Liaw H, Woodgate R, Park JH, et al. SHPRH regulates rRNA transcription by recognizing the histone code in an mTOR-dependent manner. Proc Natl Acad Sci. 2017;114:E3424–E33. 28400511 10.1073/pnas.1701978114 PMC5410823

[77] Buckley AR, Ideker T, Carter H, Harismendy O, Schork NJ. Exome-wide analysis of bi-allelic alterations identifies a Lynch phenotype in The Cancer Genome Atlas. Genome Med. 2018;10:69. 30217226 10.1186/s13073-018-0579-5 PMC6138910

[78] van Toorn M, Turkyilmaz Y, Han S, Zhou D, Kim HS, Salas-Armenteros I, et al. Active DNA damage eviction by HLTF stimulates nucleotide excision repair. Mol Cell. 2022;82:1343–58.e8. 35271816 10.1016/j.molcel.2022.02.020 PMC9473497

[79] Lundin C, North M, Erixon K, Walters K, Jenssen D, Goldman AS, et al. Methyl methanesulfonate (MMS) produces heat-labile DNA damage but no detectable in vivo DNA double-strand breaks. Nucleic Acids Res. 2005;33:3799–811. 16009812 10.1093/nar/gki681 PMC1174933

[80] Motegi A, Sood R, Moinova H, Markowitz SD, Liu PP, Myung K. Human SHPRH suppresses genomic instability through proliferating cell nuclear antigen polyubiquitination. J Cell Biol. 2006;175:703–8. 17130289 10.1083/jcb.200606145 PMC2064669

[81] Motegi A, Liaw HJ, Lee KY, Roest HP, Maas A, Wu X, et al. Polyubiquitination of proliferating cell nuclear antigen by HLTF and SHPRH prevents genomic instability from stalled replication forks. Proc Natl Acad Sci USA. 2008;105:12411–6. 18719106 10.1073/pnas.0805685105 PMC2518831

[82] Lin J-R, Zeman MichelleK, Chen J-Y, Yee M-C, Cimprich KarleneA. SHPRH and HLTF Act in a Damage-Specific Manner to Coordinate Different Forms of Postreplication Repair and Prevent Mutagenesis. Mol Cell. 2011;42:237–49. 21396873 10.1016/j.molcel.2011.02.026 PMC3080461

[83] Tomi NS, Davari K, Grotzky D, Loos F, Böttcher K, Frankenberger S, et al. Analysis of SHPRH functions in DNA repair and immunoglobulin diversification. DNA Repair. 2014;24:63–72. 25311267 10.1016/j.dnarep.2014.09.010

[84] Frankum J, Moudry P, Brough R, Hodny Z, Ashworth A, Bartek J, et al. Complementary genetic screens identify the E3 ubiquitin ligase CBLC, as a modifier of PARP inhibitor sensitivity. Oncotarget. 2015;6:10746–58. 25883215 10.18632/oncotarget.3628 PMC4484416

[85] Dasari S, Tchounwou PB. Cisplatin in cancer therapy: molecular mechanisms of action. Eur J Pharm. 2014;740:364–78. 10.1016/j.ejphar.2014.07.025 PMC4146684 25058905

[86] Fennell DA, Porter C, Lester J, Danson S, Blackhall F, Nicolson M, et al. Olaparib maintenance versus placebo monotherapy in patients with advanced non-small cell lung cancer (PIN): A multicentre, randomised, controlled, phase 2 trial. EClinicalMedicine. 2022;52:101595. 35990583 10.1016/j.eclinm.2022.101595 PMC9386392

[87] Politi K, Herbst RS. Lung cancer in the era of precision medicine. Clin Cancer Res. 2015;21:2213–20. 25979927 10.1158/1078-0432.CCR-14-2748 PMC4505624

[88] Lawrence MS, Stojanov P, Polak P, Kryukov GV, Cibulskis K, Sivachenko A, et al. Mutational heterogeneity in cancer and the search for new cancer-associated genes. Nature. 2013;499:214–8. 23770567 10.1038/nature12213 PMC3919509

[89] Tokheim CJ, Papadopoulos N, Kinzler KW, Vogelstein B, Karchin R. Evaluating the evaluation of cancer driver genes. Proc Natl Acad Sci USA. 2016;113:14330–5. 27911828 10.1073/pnas.1616440113 PMC5167163

[90] Sood R, Makalowska I, Galdzicki M, Hu P, Eddings E, Robbins CM, et al. Cloning and characterization of a novel gene, SHPRH, encoding a conserved putative protein with SNF2/helicase and PHD-finger domains from the 6q24 region. Genomics. 2003;82:153–61. 12837266 10.1016/S0888-7543(03)00121-6

[91] Lhotova K, Stolarova L, Zemankova P, Vocka M, Janatova M, Borecka M, et al. Multigene Panel Germline Testing of 1333 Czech Patients with Ovarian Cancer. Cancers. 2020;12:956. 32295079 10.3390/cancers12040956 PMC7226062

[92] Qu Y, Gharbi N, Yuan X, Olsen JR, Blicher P, Dalhus B, et al. Axitinib blocks Wnt/beta-catenin signaling and directs asymmetric cell division in cancer. Proc Natl Acad Sci USA. 2016;113:9339–44. 27482107 10.1073/pnas.1604520113 PMC4995957

[93] Zhang M, Huang N, Yang X, Luo J, Yan S, Xiao F, et al. A novel protein encoded by the circular form of the SHPRH gene suppresses glioma tumorigenesis. Oncogene. 2018;37:1805–14. 29343848 10.1038/s41388-017-0019-9

[94] Ju X, Tang Y, Qu R, Hao S. The Emerging Role of Circ-SHPRH in Cancer. Onco Targets Ther. 2021;14:4177–88. 34285509 10.2147/OTT.S317403 PMC8286153

[95] Wong MP, Fung LF, Wang E, Chow WS, Chiu SW, Lam WK, et al. Chromosomal aberrations of primary lung adenocarcinomas in nonsmokers. Cancer. 2003;97:1263–70. 12599234 10.1002/cncr.11183

[96] Rutherford S, Yu Y, Rumpel CA, Frierson HF Jr, Moskaluk CA. Chromosome 6 deletion and candidate tumor suppressor genes in adenoid cystic carcinoma. Cancer Lett. 2006;236:309–17. 16054751 10.1016/j.canlet.2005.05.049

[97] Kluth M, Jung S, Habib O, Eshagzaiy M, Heinl A, Amschler N, et al. Deletion lengthening at chromosomes 6q and 16q targets multiple tumor suppressor genes and is associated with an increasingly poor prognosis in prostate cancer. Oncotarget. 2017;8:108923–35. 29312579 10.18632/oncotarget.22408 PMC5752492

[98] Amos CI, Pinney SM, Li Y, Kupert E, Lee J, de Andrade MA, et al. A susceptibility locus on chromosome 6q greatly increases lung cancer risk among light and never smokers. Cancer Res. 2010;70:2359–67. 20215501 10.1158/0008-5472.CAN-09-3096 PMC2855643

[99] Chen EG, Chen Y, Dong LL, Zhang JS. Effects of SASH1 on lung cancer cell proliferation, apoptosis, and invasion in vitro. Tumour Biol. 2012;33:1393–401. 22488244 10.1007/s13277-012-0387-2

[100] You M, Wang D, Liu P, Vikis H, James M, Lu Y, et al. Fine mapping of chromosome 6q23-25 region in familial lung cancer families reveals RGS17 as a likely candidate gene. Clin Cancer Res. 2009;15:2666–74. 19351763 10.1158/1078-0432.CCR-08-2335 PMC2746091

[101] Wilson IM, Vucic EA, Enfield KS, Thu KL, Zhang YA, Chari R, et al. EYA4 is inactivated biallelically at a high frequency in sporadic lung cancer and is associated with familial lung cancer risk. Oncogene. 2014;33:4464–73. 24096489 10.1038/onc.2013.396 PMC4527534

[102] Unk I, Hajdu I, Blastyak A, Haracska L. Role of yeast Rad5 and its human orthologs, HLTF and SHPRH in DNA damage tolerance. DNA Repair. 2010;9:257–67. 20096653 10.1016/j.dnarep.2009.12.013

