# Supplementary material for: Integrative genomics identifies SHPRH as a tumor suppressor gene in lung adenocarcinoma that regulates DNA damage response
Source: Br J Cancer. 2024 Jun 18;131(3):534–50. doi: 10.1038/s41416-024-02755-y (PMC11300780; doi:10.1038/s41416-024-02755-y)
Supplement: Supplementary file 1 — Supplemental Figures and Legends [file 41416_2024_2755_MOESM1_ESM.pdf]

Supplemental Figure 1

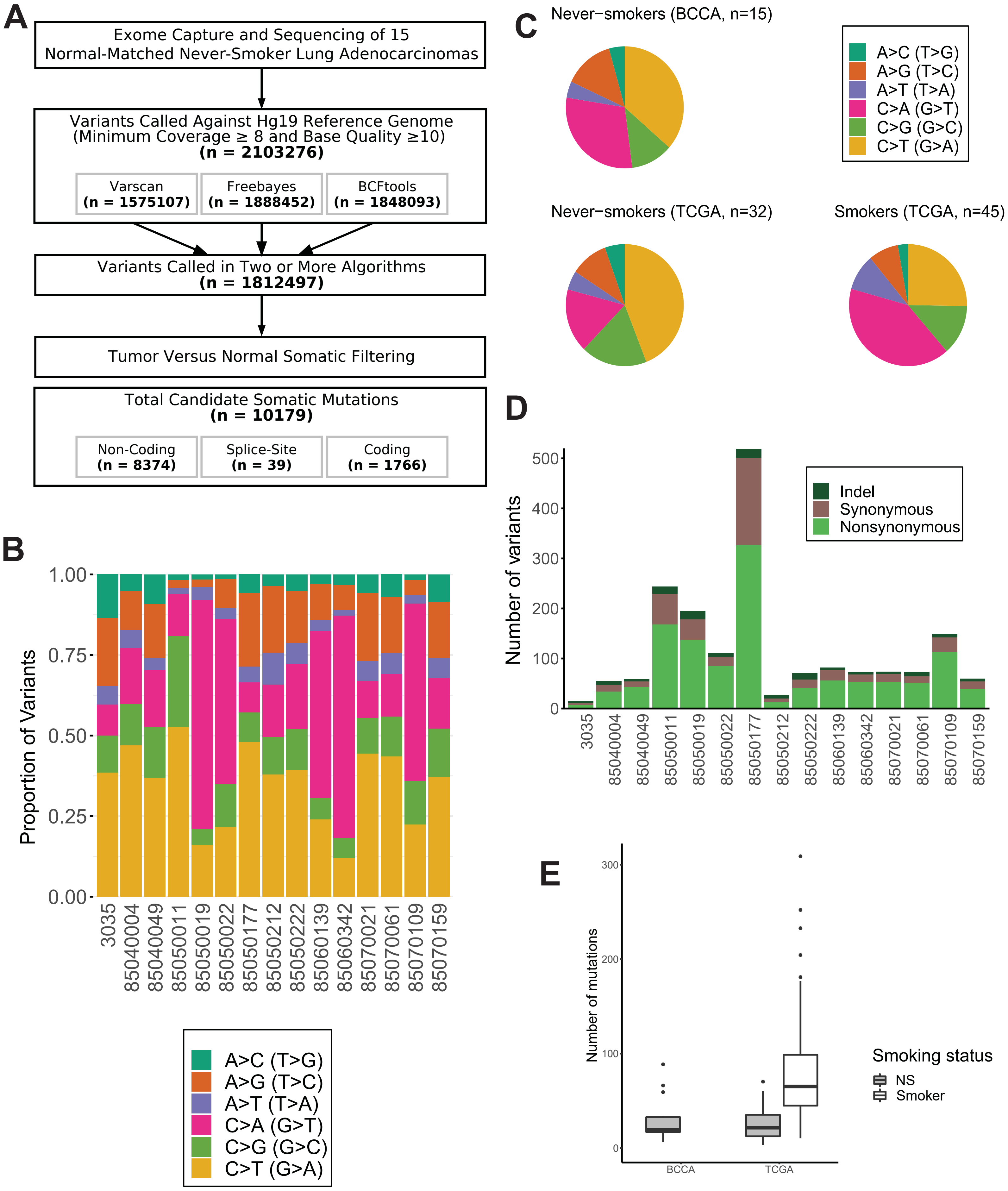

Supplemental Figure 2

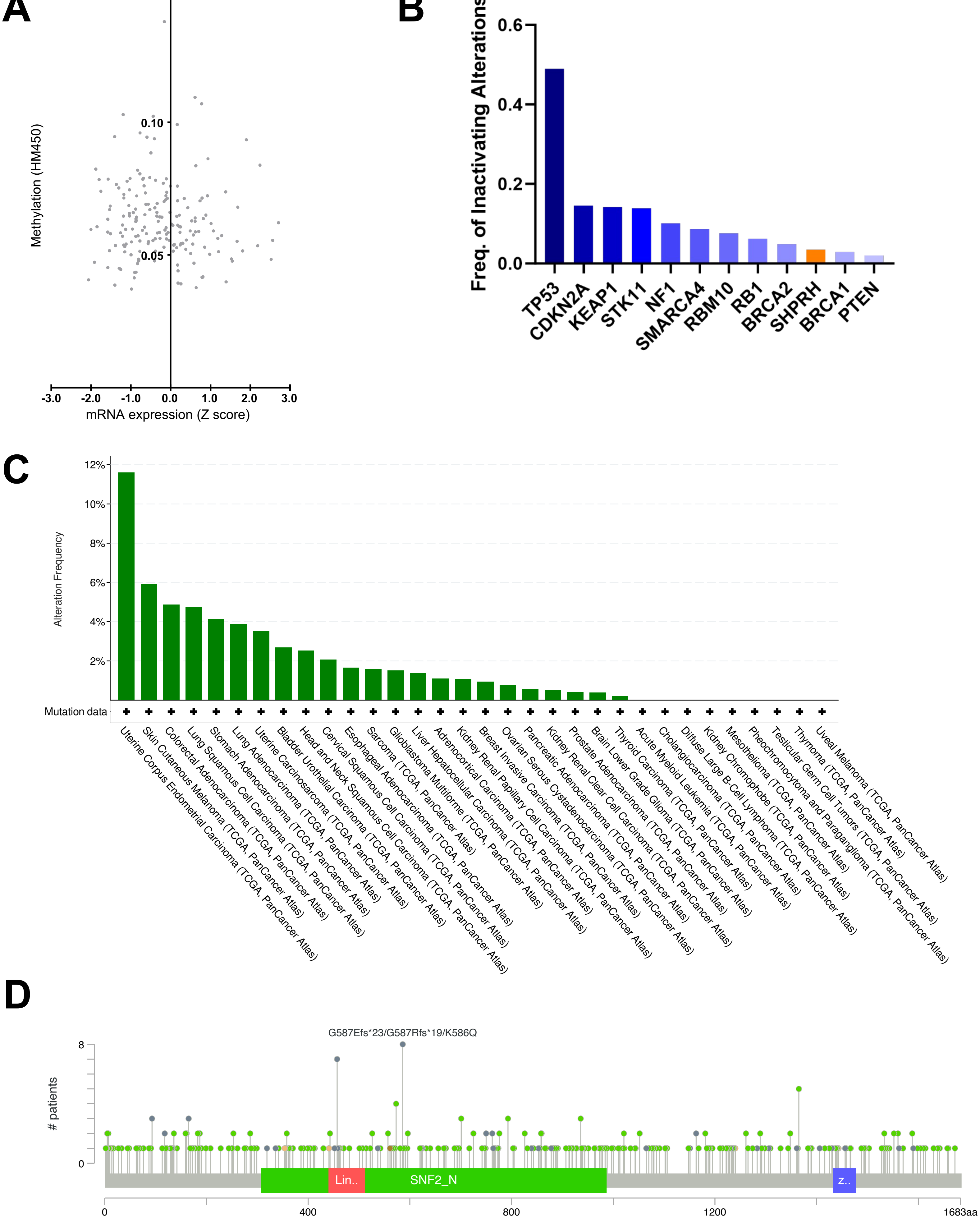

Supplemental Figure 3

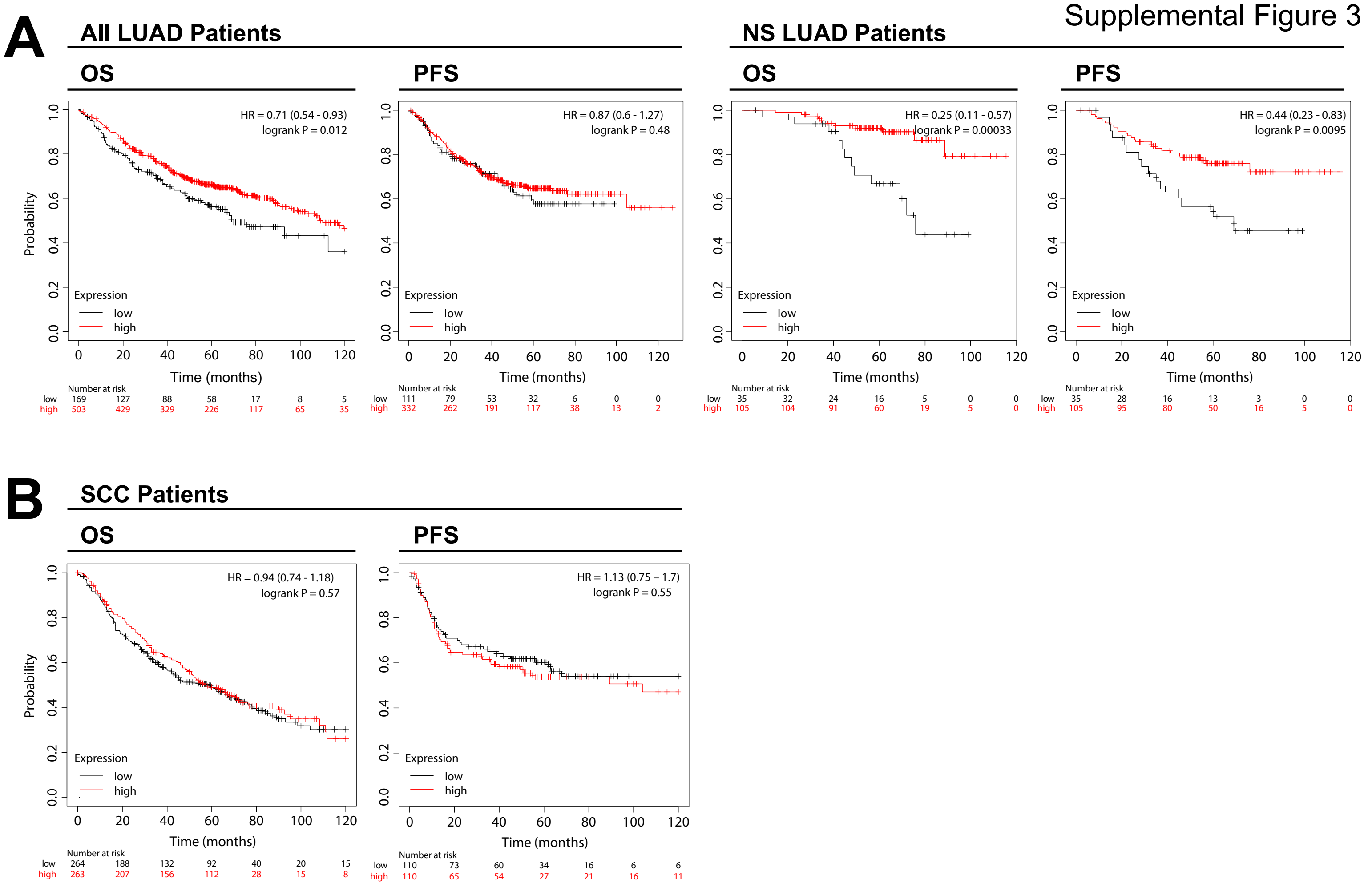

A

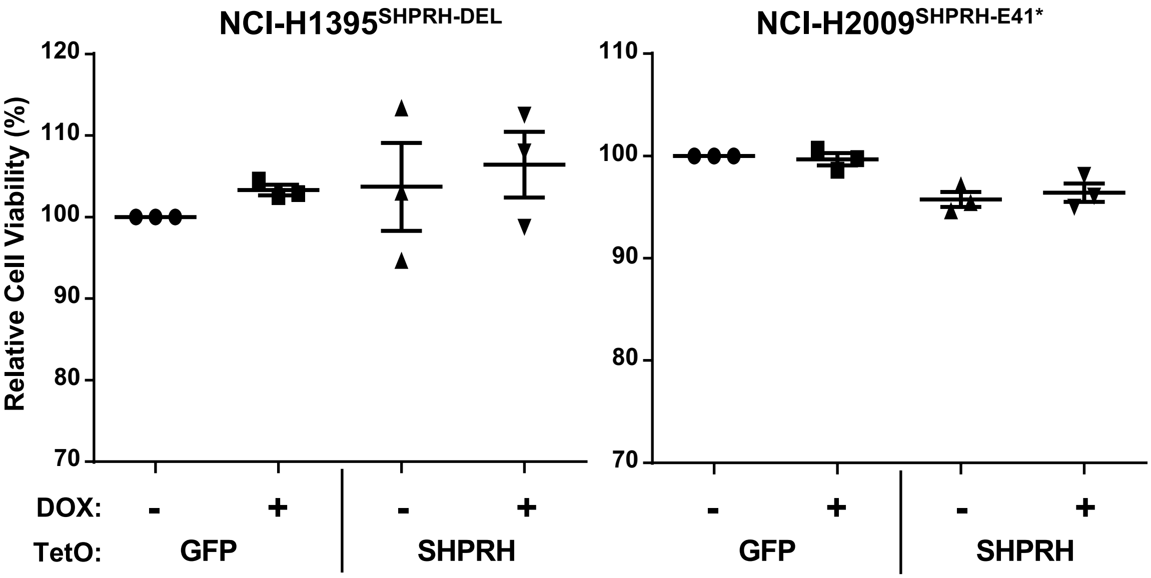
NCI-H1395SHPRH-DEL
NCI-H2009SHPRH-E41*
Relative Cell Viability (%)
120
110
100
90
80
70
110
100
90
80
70
DOX:
-
+
-
+
-
+
-
+
TetO:
GFP
SHPRH
GFP
SHPRH

Supplemental Figure 5

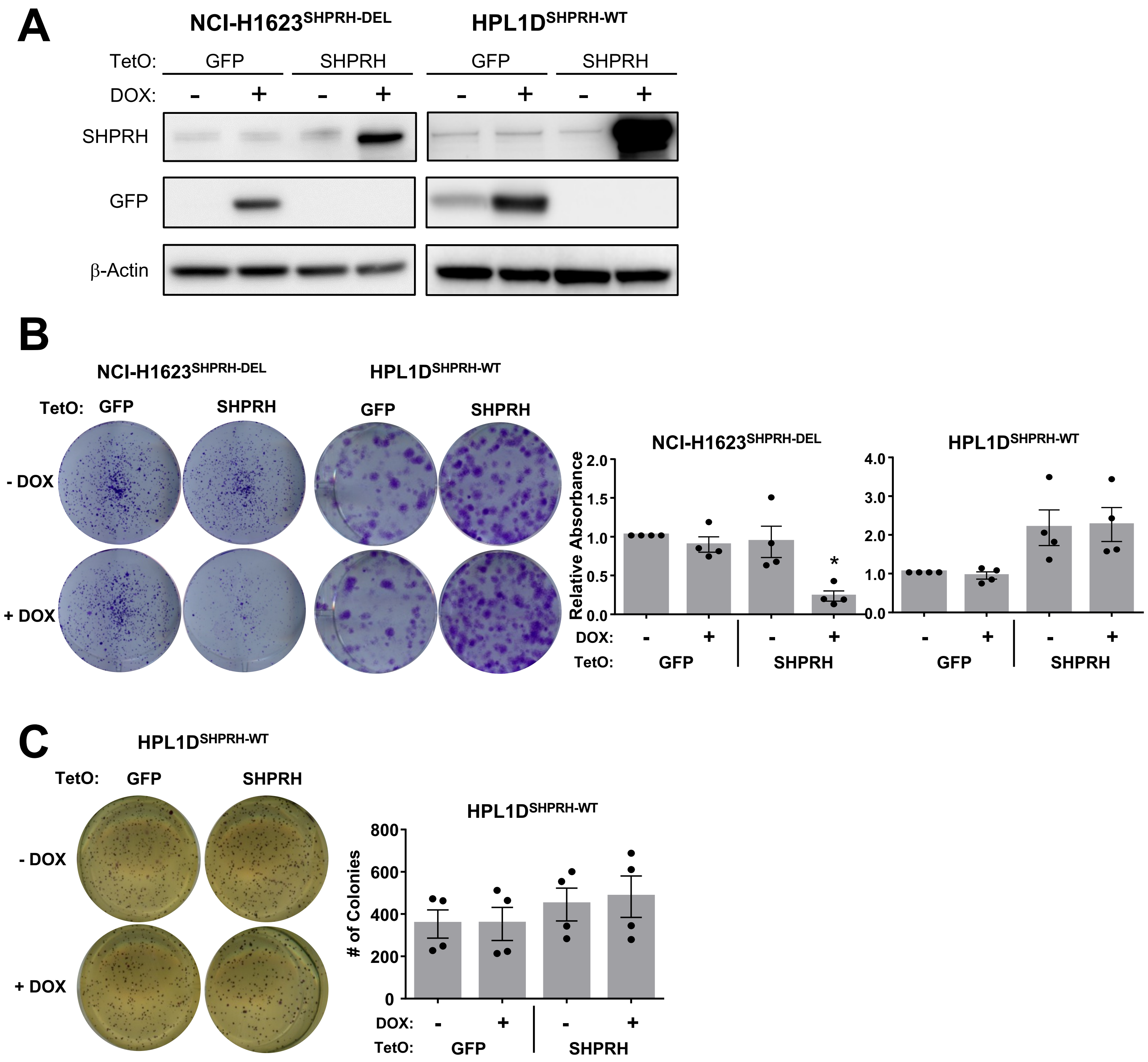

A

NCI-H1395$^{SHPRH-DEL}$

Normal Diet

TetO GFP

TetO SHPRH

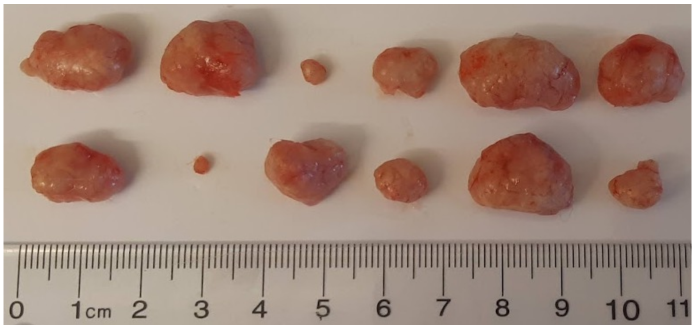

Dox Diet

TetO GFP

TetO SHPRH

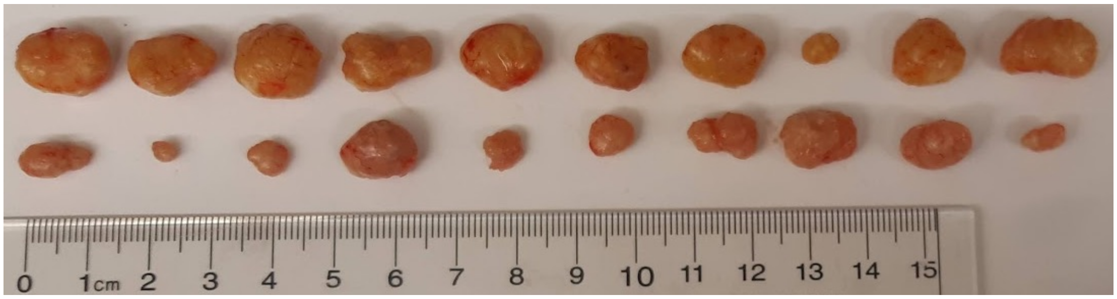

NCI-H2009$^{SHPRH-E41*}$

Normal Diet

TetO GFP

TetO SHPRH

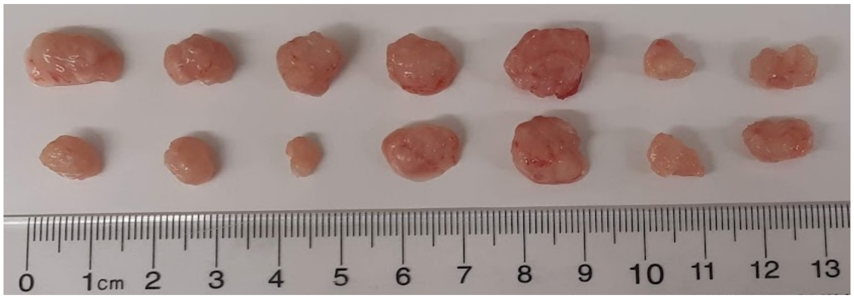

Dox Diet

TetO GFP

TetO SHPRH

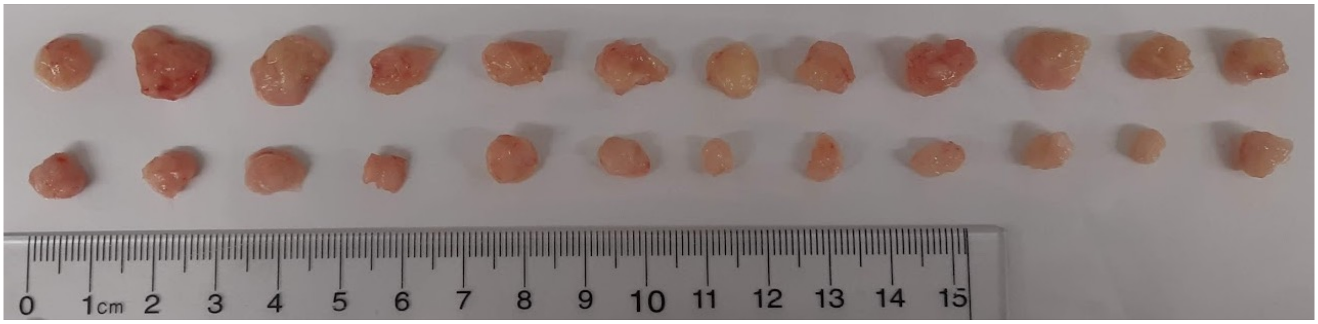

A NCI-H1395^SHPRH-DEL

Normal Diet

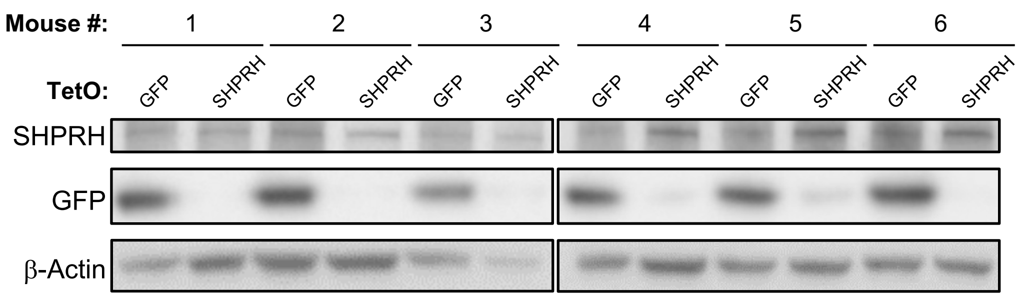

Dox Diet

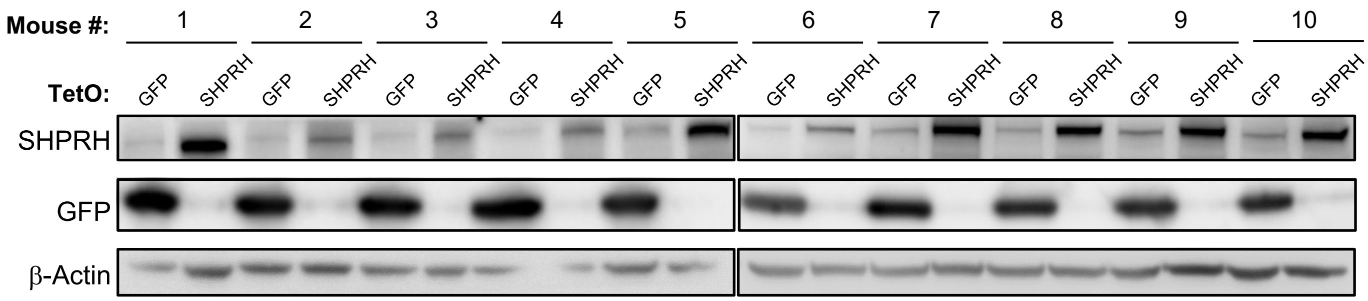

NCI-H2009^SHPRH-E41*

Normal Diet

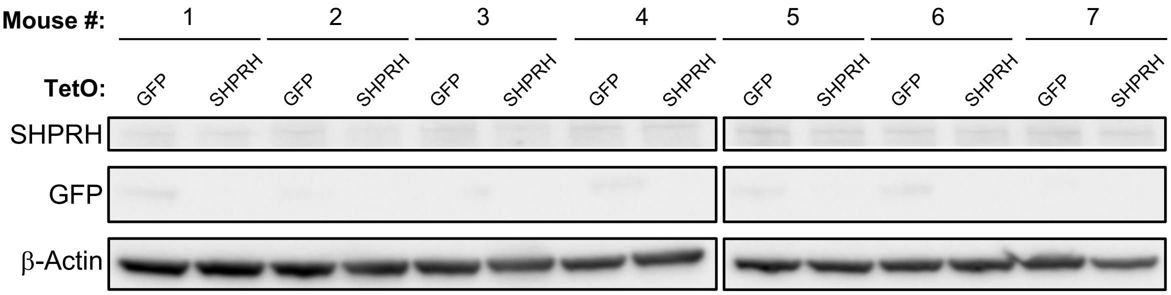

Dox Diet

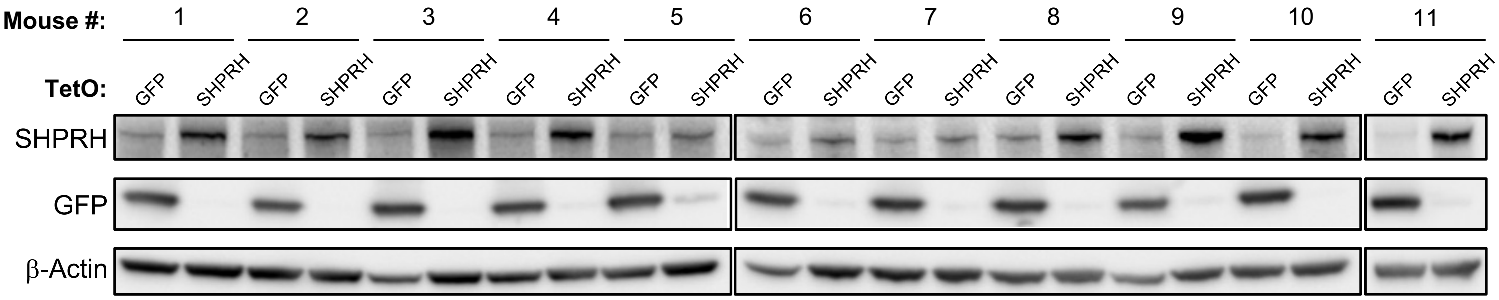

Supplemental Figure 8

A

Vector	sgRNA sequence
lentiCRISPRv2-EV	–
lentiCRISPRv2-sgSHPRH.ex4	ATGCTGGACATCCACTTGGA

B

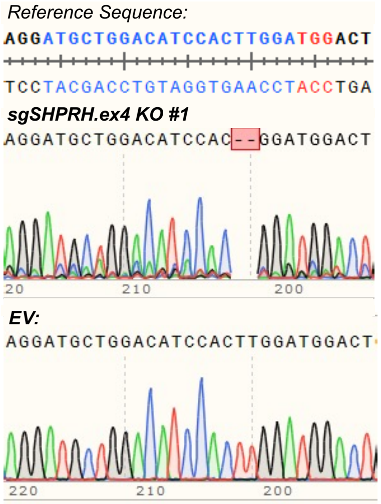

C

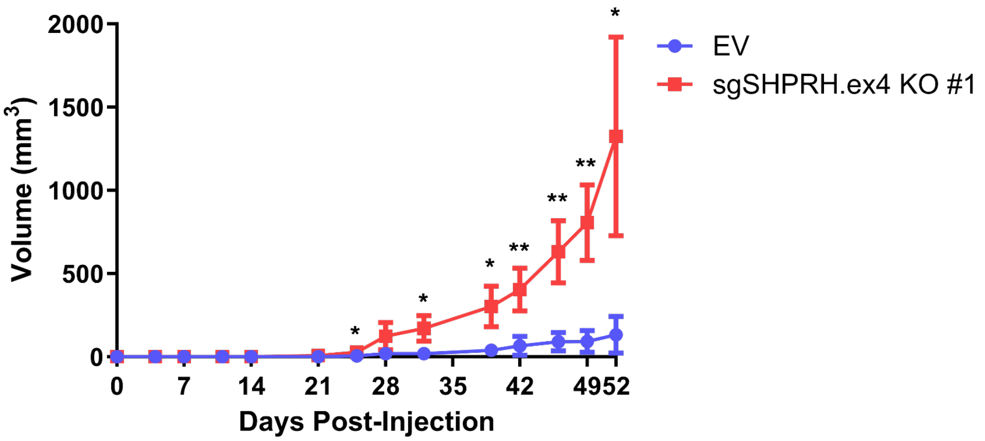

D

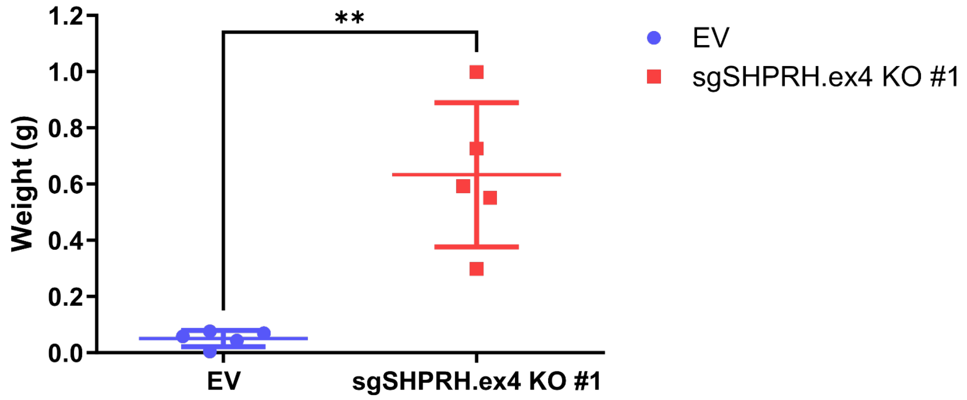

E

EV sgSHPRH.ex4 KO #1

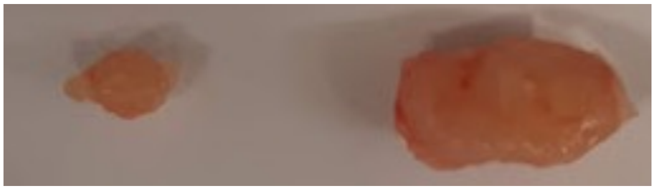

F

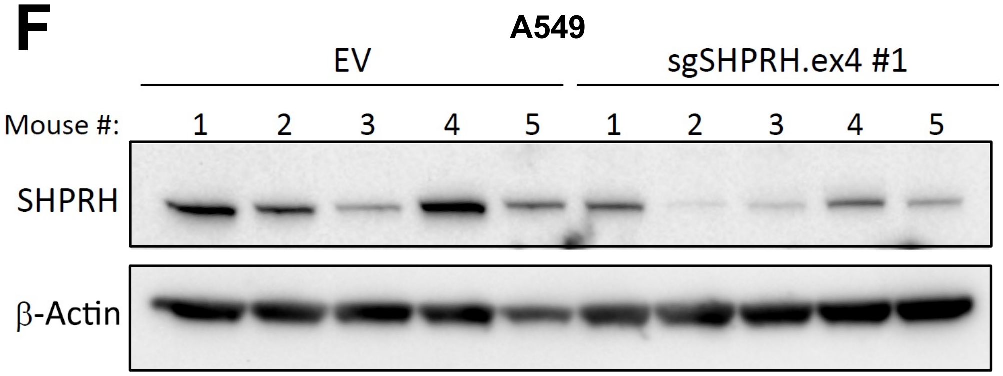

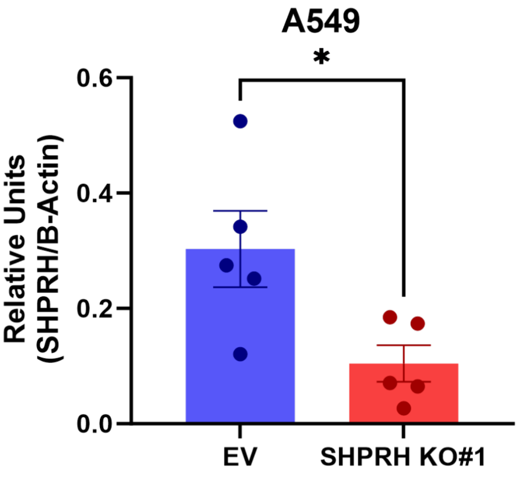

Supplemental Figure 9

A

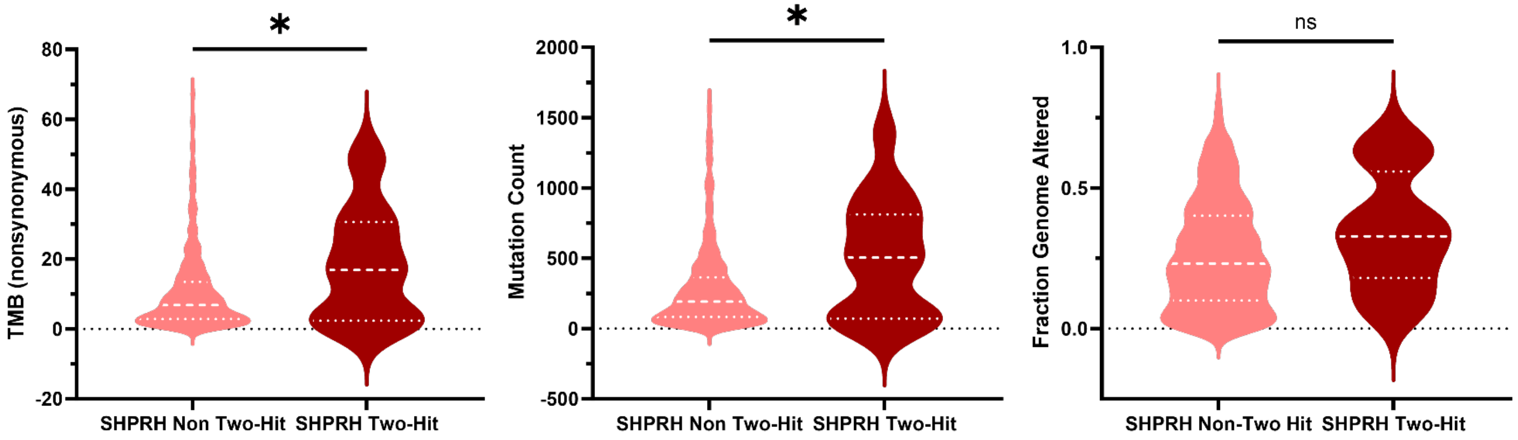

Supplemental Figure 1. WES calling pipeline and somatic variant characteristics of 15 NS LUAD tumors. A) Sequencing workflow used for identifying somatic mutations. Low quality variants present in 2 of the 3 variant-calling algorithms were retained, while variants called in any normal control sample were removed. B) Mutational spectrum showing the proportion of variants corresponding to given transitions/transversions type across the 15 tumors. C) Pie chart indicating the fraction of transitions and transversions called across this dataset (BCCA, top) and smokers and non-smokers in the publicly available TCGA dataset (TCGA, bottom). D) Mutation distribution density of coding variant frequencies across the 15 tumors. E) Box- and-whisker plot showing number of mutations present across BCCA and TCGA cohorts with smokers and non-smokers segregated. Number of mutations for both BCCA and TCGA corresponds to post-functional filtering represented in Figure 1.

Supplemental Figure 2. A) Scatter plot showing the association between methylation status and mRNA expression of *SHPRH* in LUAD tumors (n=74). B) Frequency of homozygous deletion or mutation in *SHPRH* and 11 well-characterized LUAD tumor suppressor genes in 980 LUADs with matching sequence and copy number data from four LUAD datasets from the cBioPortal (LUAD Broad Cell 2012, LUAD CPTAC Cell 2020, LUAD TCGA PanCan Atlas, LUAD OncoSG Nat Genet 2020), L. C) Frequency of inactivating *SHPRH* alterations across 32 different cancer types from the TCGA PanCancer Atlas dataset in cBioPortal. D) Lollipop plot of mutation sites across the SHPRH gene in the PanCancer Atlas dataset in cBioPortal.

Supplemental Figure 3. A) KM Plots showing OS and PFS outcomes in LUAD patients (n=672 for OS, n=528 for PFS) and NS LUAD patients (n=140). Patients are lower quartile split into

SHPRH low (black) or high (red) expression. Logrank P-values and HRs from the cox univariate regression model are shown. C) KM Plots showing overall survival (OS) and progression-free survival (PFS) outcomes in SCC patients (n=527 for OS, n= 220 for PFS). Patients are median split into SHPRH low (black) or high (red) expression. Logrank P-values and HRs from the cox univariate regression model are shown.

Supplemental Figure 4. A) AlamarBlue viability results of NCI-H1395$^{SHPRH\text{-}DEL}$ and NCI-H2009$^{SHPRH\text{-}E41*}$ TetO GFP and SHPRH cells with or without the addition of 100ng/mL dox for 96 hours. Viability is calculated relative to GFP no dox condition. Mean ± SEM of N=3 independent replicates and results from student's t-test are shown.

Supplemental Figure 5. A) Induction of SHPRH in NCI-H1623$^{SHPRH\text{-}DEL}$ (*SHPRH* homozygous deletion) and HPL1D$^{SHPRH\text{-}WT}$ (*SHPRH* wildtype) cell lines. GFP or SHPRH was induced by the addition of 100ng/mL of dox and protein levels were measured by Western blot. B) Left: Representative images of clonogenic plates showing differences in anchorage-dependent colony growth in TetO GFP and SHPRH cells with or without the addition of 100ng/mL dox. Right: Quantification of crystal violet staining, calculated relative to GFP no dox condition. Mean ± SEM of N=4 independent replicates is shown. Results from student's t-test are indicated, where *p≤0.05. C) Left: Representative images of soft agar plates showing anchorage-independent colony formation in TetO GFP and SHPRH cells with or without the addition of 100ng/mL dox. Right: Quantification of colonies. Mean ± SEM of N=4 independent replicates and results from student's t-test are shown.

Supplemental Figure 6. A) Images of all NCI-H1395$^{SHPRH\text{-}DEL}$ (above) and NCI-H2009$^{SHPRH\text{-}E41*}$ (below) TetO GFP and SHPRH tumors harvested from mice kept on a normal (N=6 mice for NCI-H1395; N=7 mice for NCI-H2009) or dox diet (N=10 mice for NCI-H1395; N=11 mice for NCI-H2009).

Supplemental Figure 7. A) Western blot validation of GFP and SHPRH expression in NCI-H1395$^{SHPRH\text{-}DEL}$ (above) and NCI-H2009$^{SHPRH\text{-}E41*}$ (below) tumors harvested from mice kept on a normal (N=6 mice for NCI-H1395; N=7 mice for NCI-H2009) or dox diet (N=10 mice for NCI-H1395; N=11 mice for NCI-H2009).

Supplemental Figure 8. A) sgRNA sequences inserted into lentiCRISPRv2 to target either exon 4 of SHPRH (-sgSHPRH.ex4) or used as an empty vector (EV) control. B) Representative Sanger sequencing chromatograms validating CRISPR/Cas9-treated monoclonal populations. Sequences were aligned to the EV control to detect for indels. sgRNA sequence (blue nucleotides), PAM sequence (red nucleotides), and deletions (red box) are labelled. C) Tumour volume, D) weight measurements, and E) representative tumour images of A549 EV and sgSHPRH.ex4 KO #1 cells subcutaneously injected in alternating flanks of N=5 NRG mice. Results from student's paired t-test are indicated, where *$p<0.05$ and **$p\leq0.01$. F) *Left*: Western validation of SHPRH KO status in tumours harvest from mice implanted with A549 EV and sgSHPRH.ex4 KO #1 cells. *Right:* Quantification of SHPRH protein density, displayed relative to β-Actin. Mean ± SEM of N=5 of individual tumours is shown and results from paired t-tests are indicated, where *$p<0.05$ and **$p\leq0.01$.

Supplemental Figure 9. Violin plots outlining the Tumour Mutation Burden (*Left*), Mutation Count (*Center*), and Fraction of Genome Altered (*Right*) in LUAD cases with either one-hit or two-hit inactivating alterations of *SHPRH*. Results from one-tailed Mann-Whitney U tests are shown, where *$p \leq 0.05$ and ** $p \leq 0.01$
